# The hospital admission profile of people presenting to specialist addiction services with problematic use of alcohol or opioids: A national retrospective cohort study in England

**DOI:** 10.1016/j.lanepe.2021.100036

**Published:** 2021-04

**Authors:** Emmert Roberts, Matthew Hotopf, John Strang, John Marsden, Martin White, Brian Eastwood, Colin Drummond

**Affiliations:** a National Addiction Centre and the Department of Psychological Medicine, Institute of Psychiatry, Psychology and Neuroscience, King's College London, South London and the Maudsley NHS Foundation Trust and Public Health England, United Kingdom; b Department of Psychological Medicine, Institute of Psychiatry, Psychology and Neuroscience, King's College London and South London and the Maudsley NHS Foundation Trust, United Kingdom; c National Addiction Centre, Institute of Psychiatry, Psychology and Neuroscience, King's College London and South London and the Maudsley NHS Foundation Trust, United Kingdom; d National Addiction Centre, Institute of Psychiatry, Psychology and Neuroscience, King's College London and South London and the Maudsley NHS Foundation Trust, United Kingdom; e Alcohol, Drugs, Tobacco and Justice division, Public Health England, United Kingdom; f National Addiction Centre, Institute of Psychiatry, Psychology and Neuroscience, King's College London and South London and the Maudsley NHS Foundation Trust, United Kingdom

## Abstract

**Background:**

Over the past decade in England the rate of alcohol and opioid-related hospitalisation has increased alongside a simultaneous reduction in people accessing specialist addiction treatment. We aimed to determine the hospitalisation patterns of people presenting to addiction treatment with problematic use of alcohol or opioids, and estimate how individual sociodemographic characteristics and hospital admission diagnoses are associated with the rate of hospitalisation, death and successful completion of addiction treatment.

**Methods:**

A national record linkage between Hospital Episode Statistics (HES) and the National Drug Treatment Monitoring System (NDTMS) captured lifetime hospital admission profiles of people presenting to addiction services in England in 2018/19. Latent class analysis assigned individuals to clusters based on the ICD-10 diagnosis coded as primary reason for admission. Negative binomial, and multilevel logistic regression models determined if outcomes differed due to sociodemographic characteristics or assigned diagnostic clusters.

**Findings:**

Inpatient data were available for 64,840 alcohol patients, and 107,296 opioid patients. The most common reasons for admission were alcohol withdrawal (*n* = 20,024 (5.3% of alcohol-cohort admissions)), and unspecified illness (*n* = 11,387 (2.1% of opioid-cohort admissions)). Seven diagnostic clusters were identified for each substance cohort. People with admissions predominantly relating to mental and behavioural disorders, and injuries or poisonings had significantly higher hospitalisation rates (adjusted IRR 7.06 (95%CI 6.72–7.42);*p* < 0.001), higher odds of death during addiction treatment (adjusted OR 2.71 (95%CI 2.29–3.20);*p* < 0.001) and lower odds of successful treatment completion (adjusted OR 0.72 (95%CI 0.68–0.76);*p* < 0.001).

**Interpretation:**

This is the first study to interrogate national hospitalisation patterns within people presenting to addiction services with problematic use of alcohol or opioids. Having identified high-risk, high-cost individuals with increased hospital usage, and increased odds of death, future work should focus on targeting appropriate interventions, to improve their health outcomes and prevent unnecessary hospital readmission.

**Funding:**

The work was funded by the Medical Research Council (MRC).

**Research in context**

**Evidence before this study**

Alcohol and non-medical opioid use are associated with a substantial global burden of disease, and are responsible for millions of inpatient hospital admissions per year. Previous research reports rates of inpatient hospital admission two to eight times higher in individuals with substance use disorders when compared to groups without substance misuse diagnoses.[1] However, these estimates are largely based subsamples of people identified at the level of individual services, or with specific conditions. To our knowledge only one previous study in Scotland reports national level hopsitalisation estimates for the cohort of people with problematic alcohol or opioid use presenting to specialist addiction services. Whilst this study reports significantly elevated admission rates for diagnoses pertaining to mental and behavioural disorders, it does not identify groups of people who may be disproportionally responsible for elevated admission rates.[2]

**Added value of this study**

With the creation of a novel data-linkage, we believe this is the largest study globally to be able to interrogate national hospitalisation patterns within the cohort of people presenting to specialist addiction services with problematic use of alcohol or opioids. For the first time this paper characterises distinct high-risk high-cost clinical cohorts with hospital admission diagnoses predominantly related to mental and behavioural disorders, and injuries or poisonings that have dramatically increased rates of hopsitalisation (adjusted Incidence Rate Ratios (aIRRs) of 7.06 95%CI 6.72–7.42, *p* < 0.001 for alcohol, and 7.50 95%CI 7.11–7.9, *p* < 0.001 for opioids), and an associated increased odds of mortality (adjusted Odds Ratios (aORs) of 2.71 95%CI 2.29–3.20, *p* < 0.001 for alcohol, and 2.11 95%CI 1.90–2.35, *p* < 0.001 for opioids).

**Implications of all the available evidence**

Given the global harm associated with alcohol, and non-medical opioid use, this study is able to suggest which people, at the point at which they present for addiction treatment, are at high risk of hospitalisation, high odds of death during treatment and low odds of successful completion of specialist addiction treatment. As such future work within these cohorts should focus on the development of targeted interventions to reduce unnecessary hospital readmission, and improve health outcomes.

Alt-text: Unlabelled box

## 1 Introduction

Alcohol and non-medical opioid use are modifiable risk factors associated with substantial multimorbidity, and are responsible for millions of preventable deaths and hospital admissions worldwide each year [3,4]. In financial year 2018/19 over 80% of people presenting to specialist addiction services in England attended due to problematic use of alcohol or opioids [5], and there were an estimated 1.3 million hospital admissions related to alcohol, an increase of 15% compared to the previous decade [6,7]. Deaths due to opioid overdose have more than doubled over the same timeframe [8], this increasing rate of substance-related harm being particularly pertinent in England given the recent substantial financial disinvestment in specialist addiction services [9], and a concomitant decrease in the number of people accessing addiction treatment. Between 2013/14 and 2018/19, reductions of 12% and 7% have been recorded for alcohol and opioid patients, respectively [5,10].

In a 2016 report funded by the English Department of Health identified an urgent need to estimate the impact of specialist addiction treatment on acute care resource usage and substance-related harm [11]. The report posited this may be achieved through detailed analysis of linked individual-level hospitalisation and substance misuse treatment data, which would be able to ‘…*generate evidence to quantify the impact on health service utilisation”* [11]. Until now an understanding of the hospitalisation patterns of individuals presenting to specialist addiction services has been hindered due to the lack of available linked data, with separate database systems in England currently capturing the national activity on hospitalisation and addiction treatment. Whilst international efforts have been made to facilitate record linkage of national databases in order to evaluate substance misuse outcomes [12], previous studies have often lacked access to national level data on substance misuse treatment, due in part to fragmented healthcare delivery systems or lack of a centralised data repository.

A novel record linkage, completed in 2020 by Public Heath England (PHE), between the Hospital Episode Statistics (HES) Admitted Patient Care (APC) database and the National Drug Treatment Monitoring System (NDTMS) [13], for the first time provides a resource that is able to determine the reasons for, and frequency of, inpatient hospital admissions among the cohort of people accessing specialist addiction treatment in England. We aimed to interrogate this linked dataset to characterise hospitalisation patterns within this cohort, describe the main diagnostic reasons for admission, and estimate how individual sociodemographic and clinical risk factors impact the rate of inpatient hospital admission, the odds of death during specialist addiction treatment and the odds of successfully completing specialist addiction treatment.

## 2 Methods

### 2.1 Data sources

The National Drug Treatment Monitoring System (NDTMS) is the centralised database for specialist addiction treatment and is collated and maintained by PHE. NDTMS receives monthly input from all local authority commissioned and publicly funded community addiction services in England [14]. It contains data on an individual's sociodemographic characteristics (date of birth, gender, ethnicity, housing status etc.), the interventions received, whether the individual died during treatment and measures of treatment success. Individuals within NDTMS are categorised based on the substances they report using problematically, with data used herein from patients who, at presentation, report alcohol as the only substance which they use problematically and patients who, at presentation, report any problematic use of opioids [14]. Data for this study was included from unique individuals presenting to addiction services in England in financial year 2018/19.

Hospital Episode Statistics (HES) is the centralised repository, collated and maintained by NHS Digital, which collects all information pertaining to National Health Service (NHS) hospitalisation in England and Wales [15]. The HES Admitted Patient Care (APC) database is one of the main administrative databases operating under the umbrella of HES and covers all NHS inpatient admissions, including any admission to private or third sector hospitals subsequently reimbursed by the NHS [16]. As such, HES APC is estimated to contain >99% of all inpatient hospital activity in England [17]. An inpatient hospital admission includes any secondary care-based activity requiring a hospital bed, thus includes day cases, births and deliveries, and both elective and emergency admissions, in physical and mental health settings. HES APC does not cover accident and emergency (A&E) attendances nor outpatient bookings, these data being held in separate HES databases. Hospital admission records are available since HES database inception on 1st April 1997.

People accessing publicly-funded specialist addiction treatment services in England provide written consent to share their information with NDTMS and are informed that NDTMS records may be linked with data from other specifically sanctioned UK government-held databases, including HES [18]. Over 98% of patients provide consent [19], and the nature of this consent states that individuals may opt out at any time from having their records used. Approval to conduct the linkage analysis was granted under regulation 3 of the Health Service (Control of Patient Information) Regulations 2002 [20], following review by the PHE Caldicott Advisory Panel (CAP) (Ref: CAP-2019-06). The study benefited throughout from discussions with service users from the South London and the Maudsley (SLaM) Biomedical Research Centre (BRC) Data Linkage Service User and Carer Advisory Group, and the PHE Alcohol Treatment Expert Group, and is reported according to the STROBE statement [21]. There were no deviations from the study protocol, the analysis was not pre-registered, and the results should be considered exploratory.

### 2.2 Hospital admission definitions

A unique hospital admission was defined as a ‘Continuous InPatient Spell’ (CIPS) according to standard methods [22,23]. A CIPS represents an individual's continuous journey from admission to discharge from inpatient hospital services regardless of any transfers between hospital consultants responsible for care within the same hospital or any transfers between different hospitals prior to discharge. Unique completed hospital admissions were subsequently characterised as ‘general inpatient admissions’ (i.e. all inpatient hospital admissions excluding day-case visits, regular day or night attenders, and admissions for births or deliveries) [24]. HES records enable one primary and up to nineteen secondary ICD-10 diagnostic codes to be recorded for each unique completed hospital admission.

### 2.3 Statistical analysis

We described and compared the number of general inpatient admissions, the mean number of admissions per person, mean length of admission, and mean age at first admission between individuals with problematic alcohol or opioid use, and how they differ by sociodemographic (sex, age, ethnicity, residential status and deprivation) and clinical (type of admission) characteristics. Continuous variables were compared using an unpaired t-test, categorical variables compared using a chi-squared test, and ordinal variables using the Wilcoxon rank sum test.

As primary reason for hospital admission is heterogeneous, we aimed to identify distinct clinical groups (i.e. diagnostic clusters) such that, within each cluster, individuals held similar patterns in their primary reason for hospital admission. Binary dummy variables were created representing each of the first 19 diagnostic chapters of ICD-10. The full list of ICD-10 diagnostic chapters, and examples of disorders from each chapter, can be found as table S1 in the online supplementary material. We used latent class analysis (LCA) to assign all individuals to non-overlapping clusters (i.e. each individual is assigned to only one cluster) [25,26]. An initial unconditional 1-class solution was fit to the data and sequentially increased to an 8-class model. Each model used 5000 random sets of starting values to guard against convergence on local maxima, and where higher order latent class models failed to converge, tolerance criteria were relaxed to generate a solution even if that solution was in a non-concave region of the parameter space. Following this, the logit intercepts for each chapter were examined and where they were <-15 or >15 these were constrained and the LCA model rerun with normal tolerance criteria. All final models converged using normal tolerance criteria. The optimal number of clusters was decided using a combination of statistical and clinical assessments (the most parsimonious model in which the expected percentage of the population in each cluster was ≥5%, the likelihood ratio test had to fail to reject the null hypothesis comparing that latent class model fit to a saturated model, the lowest combination of the Akaike's Information Criterion (AIC), and Bayesian Information Criteria (BIC), an entropy >0.8, and examination of the derived clusters to ensure they represented clinically distinct populations) [27,28]. The chosen LCA model was thus used to assign the most likely cluster membership for each individual within the cohort, and a multinomial logistic regression was used to characterise the clusters by individual-level sociodemographic characteristics. Given the hierarchical structure, with individuals clustered in treatment services and services clustered in local treatment systems, confidence intervals (CIs) were calculated using robust standard errors.

To assess the association between an individual's sociodemographic and clinical characteristics, including their LCA derived diagnostic cluster, and their hospital admission counts we used a zero-truncated negative binomial regression model to generate Incidence Rate Ratios (IRRs); an IRR greater than 1 denoting an increased rate of hospital admission when compared to the reference value. A truncated model was used as all individuals in our sample had at least one inpatient hospital admission thereby ensuring predictions were in the range of [1, ∞) rather than [0, ∞), and the negative binomial distribution was used to account for overdispersion in the number of hospital admissions. Confidence intervals were calculated with robust standard errors to account for the clustering of individuals within local authority commissioned specialist addiction treatment services. To assess the association between an individual's sociodemographic and clinical characteristics and the binary odds of death during treatment or successful treatment completion we used multilevel logistic regression models to generate Odds Ratios (ORs); an OR greater than 1 denoting an increased odds of death during treatment, and an increased odds of successful completion of treatment when compared to the reference value. Successful completion of treatment is a standard NDTMS variable based on clinic reports that the individual has reduced use or abstinence depending on treatment goals, completed interventions and met care plan goals, with mutual agreement to exit treatment [29,30]. The models were adjusted for all sociodemographic and clinical covariates and, additionally, inverse probability weighting was assigned to each individual to examine for the effect of any potential error introduced by the linkage process, as per standard methods to account for non-response bias in cohort studies [31,32]. All studied variables in the substance misuse cohorts had complete data with the exception of ethnicity and IMD, whose missingness was assumed to be completely at random (MCAR).

All analyses were conducted using SQL Server Management Studio version 18.4 and STATA SE version 15.1. The significance level was set at 0.05, no allowance was made for multiplicity.

### 2.4 Role of the funding source

This study was supported by the corresponding author's MRC Addiction Research Clinical (MARC) Fellowship. The funder had no role in study design; data collection, data analysis, interpretation of data, the writing of the report, or in the decision to submit the paper for publication. ER and BE had full access to all the data in the study and all authors had final responsibility for the decision to submit for publication.

## 3 Results

### 3.1 Characteristics of the study population

Table 1 summarises the sociodemographic and clinical profile of all studied cohorts and their hospital admissions. Linked hospitalisation data were available for 64,840 alcohol, and 107,296 opioid patients who presented to addiction services in England in 2018/19, this provided the final sample available for analysis. Since 1st April 1997 these cohorts were responsible for a total of 374,713, and 554,936 general inpatient hospital admissions respectively. When compared to the overall admission profile of all people in HES, both substance misuse cohorts had a higher mean number of admissions per person; 3.8 (all) vs 5.8 (alcohol) vs 5.2 (opioid), a lower mean length of admission 4.8 days vs 3.3 (alcohol) vs 4.3 (opioid), and a lower mean age at first admission 62.0 years (all) vs 40.8 (alcohol) vs 34.0 (opioid). When compared to the admission profile of all people in HES both substance misuse cohorts had a statistically significantly higher proportion of admissions from men, younger people, people from more deprived areas, people without a residential address, people of white ethnicities and emergency admissions.

**Table 1:** Sociodemographic and clinical characteristics of the individuals who presented to community substance misuse treatment services in England in 2018/19 and their hospital admissions since 1st April 1997.

		All HES		Alcohol		Opioid	
		Peoplen (%)	Admissionsn (%)	Peoplen (%)	Admissionsn (%)	Peoplen (%)	Admissionsn (%)
All	n (%)	41,435,198 (100.0)	157,885,932 (100.0)	64,840 (100.0)	374,713 (100.0)	107,296 (100.0)	554,936 (100.0)
	Mean number of admissions per person (n)	-	3.8	-	5.8	-	5.2
	Mean age at first admission (years)	-	62.0	-	40.8	-	34.0
	Mean length of admission (days)	-	4.8	-	3.3	-	4.3
Sociodemographic	Sex						
	Female	22,393,674 (54.0)	88,849,235 (56.3)	27,439 (42.3)	168,490 (45.0)	31,760 (29.6)	205,444 (37.0)
	Male	19,041,524 (46.0)	69,036,697 (43.7)	37,401 (57.7)	206,223 (55.0)	75,536 (70.4)	349,492 (63.0)
	Age in years (at presentation to D&A services)^1^						
	18-30	5,759,464 (13.9)	16,367,994 (10.4)	4,839 (7.5)	22,238 (5.9)	8,036 (7.5)	37,048 (6.7)
	31-45	7,799,378 (18.9)	25,577,938 (16.2)	21,856 (33.7)	120,866 (32.3)	58,631 (54.6)	299,491 (54.0)
	46-60	8,153,873 (19.7)	26,931,094 (17.1)	29,168 (45.0)	171,898 (45.9)	37,110 (34.6)	198,835 (35.8)
	60+	19,645,671 (47.5)	88,779,334 (56.3)	8,977 (13.8)	59,711 (15.9)	3,519 (3.3)	19,562 (3.5)
	Deprivation (IMD) Quintile						
	First (Most deprived)	8,708,389 (22.1)	31,628,744 (25.1)	16,953 (26.7)	111,242 (30.4)	38,059 (37.7)	210,557 (40.6)
	Second	8,471,525 (21.5)	26,742,955 (21.3)	16,756 (26.4)	97,204 (26.5)	28,628 (28.4)	146,543 (28.2)
	Third	8,103,535 (20.6)	24,622,310 (21.2)	13,604 (21.4)	74,816 (20.4)	18,024 (17.8)	86,184 (16.6)
	Fourth	7,574,304 (19.2)	22,674,817 (18.0)	10,901 (17.1)	56,612 (15.4)	11,533 (11.4)	54,851 (10.6)
	Fifth (Least deprived)	6,553,814 (16.6)	20,163,562 (16.0)	5,351 (8.4)	26,665 (7.3)	4,714 (4.7)	20,984 (4.0)
	Residential status						
	Non NFA postcode	40,986,377 (98.7)	157,204,260 (99.6)	63,908 (98.6)	368,986 (98.5)	100,235 (93.4)	514,512 (92.7)
	NFA postcode	564,090 (1.3)	681,672 (0.4)	932 (1.4)	5,727 (1.5)	7,061 (6.6)	40,424 (7.3)
	Ethnicity^2^						
	White British	25,695,526 (76.9)	95,290,213 (83.2)	56,168 (88.8)	324,508 (88.9)	92,212 (87.3)	485,581 (89.0)
	White Irish	305,176 (0.9)	964,406 (0.8)	851 (1.4)	6,482 (1.8)	1,135 (1.1)	6,027 (1.1)
	Any other White background	2,238,688 (6.7)	4,821,376 (4.2)	2,352 (3.7)	11,195 (3.1)	3,659 (3.5)	14,537 (2.7)
	White and Black Caribbean (Mixed)	96,671 (0.3)	249,795 (0.2)	296 (0.5)	1,742 (0.5)	976 (0.9)	5,711 (1.1)
	White and Black African (Mixed)	54,914 (0.2)	115,339 (0.1)	105 (0.2)	646 (0.2)	203 (0.2)	907 (0.2)
	White and Asian (Mixed)	63,992 (0.2)	144,053 (0.1)	125 (0.2)	869 (0.2)	320 (0.3)	1,477 (0.3)
	Any other mixed background	171,323 (0.5)	339,906 (0.3)	262 (0.4)	1,603 (0.4)	627 (0.6)	3,286 (0.6)
	Indian (Asian or Asian British)	772,139 (2.3)	2,360,407 (2.1)	1,002 (1.6)	6,508 (1.8)	1,090 (1.0)	4,134 (0.8)
	Pakistani (Asian or Asian British)	650,979 (1.9)	2,328,226 (2.0)	217 (0.3)	1,300 (0.3)	1,206 (1.1)	5,183 (1.0)
	Bangladeshi (Asian or Asian British)	197,879 (0.6)	620,970 (0.5)	86 (0.1)	324 (0.1)	602 (0.6)	2,346 (0.4)
	Any other Asian background	492,805 (1.5)	1,078,605 (0.9)	389 (0.6)	2,338 (0.6)	971 (0.9)	3,513 (0.6)
	Caribbean (Black or Black British)	433,619 (1.3)	1,420,414 (1.2)	372 (0.6)	2,016 (0.6)	856 (0.8)	4,527 (0.8)
	African (Black or Black British)	535,668 (1.6)	1,395,965 (1.2)	466 (0.7)	2,593 (0.71)	288 (0.3)	1,589 (0.3)
	Any other Black background	359,113 (1.1)	809,521 (0.7)	264 (0.4)	1,469 (0.4)	616 (0.6)	3,289 (0.6)
	Chinese	141,660 (0.4)	295,869 (0.3)	18 (0.03)	78 (0.02)	16 (0.02)	32 (0.01)
	Any other ethnic group	1,213,000 (3.6)	2,237,321 (2.0)	298 (0.5)	1,528 (0.4)	844 (0.8)	3,273 (0.6)
Clinical	Admission type						
	Elective	-	38,837,270 (24.7)	-	52,412 (14.0)	-	75,139 (15.0)
	Emergency	-	118,650,688 (75.3)	-	320,046 (86.0)	-	426,249 (85.0)

D&A Drug and Alcohol; IMD Indices of Multiple Deprivation; NFA No fixed abode; 1 For all people in HES age was calculated at the midpoint of 2018/19, to be comparable to age at presentation to drug and alcohol services in 2017/18 for the alcohol and opioid cohort. 2 Office of Population Censuses and Surveys (OPCS) categories A-S

### 3.2 Reasons for admission and diagnostic clusters

The ten most common primary diagnostic reasons for admission in all cohorts can be found in Table 2 with these conditions accounting for one fifth (22.2%) of all admissions for alcohol patients and one sixth (15.6%) of all admissions for opioid patients. Within the top ten primary reasons for admission, the conditions that had a statistically significantly higher proportion in the alcohol cohort compared to all admissions in HES were alcohol withdrawal state, acute alcohol intoxication, alcohol dependence, paracetamol poisoning, antidepressant poisoning and unspecified convulsions (*p* < 0.001). The conditions with a statistically significantly higher proportion in the opioid cohort were opioid dependence, paracetamol poisoning, benzodiazepine poisoning and dermatological infections (*p* < 0.001). The ten most common primary diagnostic reasons for admission in all cohorts at the level of three-digit ICD-10 code can be found in the online supplementary material as Table S2.

**Table 2:** Top ten primary reasons for general inpatient hospital admissions since 1st April 1997 of the individuals who presented to substance misuse treatment services in England in 2018/19.

Rank of Admission	Primary reason for admission	ICD-10 chapter	ICD-10 code	Admissions in substance cohortn (%)	Admissions in all HESn (%)	People who have at least one admission with this condition in substance cohortn (%)	People who have at least one admission with this condition in all HESn (%)
Alcohol							
All	All	All	All	374,713 (100.0)	157,885,932 (100.0)	64,840 (100.0)	41,435,198 (100.0)
1 (Most common)	Alcohol withdrawal state	5	F10.3	20,024 (5.3)	272,032 (0.2)	1158 (1.8)	145,556 (0.4)
2	Acute alcohol intoxication	5	F10.0	11,206 (3.0)	308,920 (0.2)	717 (1.1)	226,314 (0.5)
3	Paracetamol poisoning	19	T39.1	10,731 (2.9)	762,340 (0.5)	744 (1.1)	551,190 (1.3)
4	Alcohol dependence	5	F10.2	8,056 (2.2)	190,632 (0.1)	531 (0.8)	119,388 (0.3)
5	Chest pain, unspecified	18	R07.4	6,846 (1.8)	2,987,295 (1.9)	662 (1.0)	2,240,877 (5.4)
6	Illness, unspecified	18	R69	6,558 (1.8)	2,662,891 (1.7)	578 (0.9)	1,983,493 (4.8)
7	Abdominal pain, unspecified	18	R10.4	6,240 (1.7)	2,230,005 (1.4)	494 (0.8)	1,804,105 (4.4)
8	Other specified pregnancy related conditions	15	O26.8	4,565 (1.2)	1,941,304 (1.2)	383 (0.6)	1,246,131 (3.0)
9	Antidepressant poisoning	19	T43.2	4,346 (1.2)	212,048 (0.1)	331 (0.5)	174,636 (0.4)
10 (Least common)	Convulsions, unspecified	18	R56.8	4,120 (1.1)	545,619 (0.3)	227 (0.4)	425,967 (1.0)
Opioid							
All	All	All	All	554,936 (100.0)	157,885,932 (100.0)	107,296 (100.0)	41,435,198 (100.0)
1 (Most common)	Illness, unspecified	18	R69	11,387 (2.1)	2,662,891 (1.7)	1177 (1.1)	1,983,493 (4.8)
2	Abdominal pain, unspecified	18	R10.4	10,589 (1.9)	2,230,005 (1.4)	1091 (1.0)	1,804,105 (4.4)
3	Paracetamol poisoning	19	T39.1	9,744 (1.8)	762,340 (0.5)	802 (0.7)	551,190 (1.3)
4	Cellulitis and acute lymphangitis of other parts of limb	12	L03.1	9,078 (1.6)	1,110,911 (0.7)	928 (0.9)	847,745 (2.0)
5	Chest pain, unspecified	18	R07.4	8,719 (1.6)	2,987,295 (1.9)	1023 (1.0)	2,240,877 (5.4)
6	Opioid dependence	5	F11.2	8,625 (1.6)	43,159 (0.03)	1075 (1.0)	29,077 (0.07)
7	Other specified pregnancy related conditions	15	O26.8	7,890 (1.4)	1,941,304 (1.2)	611 (0.6)	1,246,131 (3.0)
8	Phlebitis and thrombophlebitis of other and unspecified deep vessels of lower extremities	9	I80.2	7,498 (1.4)	436,270 (0.3)	714 (0.7)	357,311 (0.9)
9	Cutaneous abscess, furuncle and carbuncle of limb	12	L02.4	6,616 (1.2)	271,368 (0.2)	791 (0.7)	223,523 (0.5)
10 (Least common)	Benzodiazepine poisoning	19	T42.4	6,473 (1.2)	176,529 (0.1)	546 (0.5)	131,550 (0.3)

ICD-10 international Classification of Diseases Volume 10

Latent class analysis identified seven diagnostic clusters as most representative of the data for each substance cohort. LCA model statistics can be found in the online supplementary tables S3 and S4, the ICD-10 chapter breakdown and a detailed description of each cluster are reported in tables S5 and S6, and the ten most common primary diagnostic reasons for admission within each cluster as tables S7 and S8. Tables 3 and 4 show the results of the multinomial logistic regression analysis of the diagnostic clusters on sociodemographic characteristics (with each cluster 1 as the referent category).

**Table 3:** Characteristics of the cohort and diagnostic sub-populations of the n=64,840 individuals who presented to community drug and alcohol treatment services in England in 2018/19 with problems related to alcohol.

		Alln (%)	AC1n (%)	AC2n (%)	AC3n (%)	AC4n (%)	AC5n (%)	AC6n (%)	AC7n (%)
All	People n (%)	64,840 (100.0)	31,132 (100.0)	10,316 (100.0)	7,350 (100.0)	5,919 (100.0)	3,594 (100.0)	3,407 (100.0)	3,122 (100.0)
	Mean number of admissions per person (n)	5.8	2.6	7.9	2.7	21.2	1.7	9.3	9.8
	Mean age at first admission (years)	40.8	40.4	39.7	29.6	44.6	32.8	31.9	47.3
	Mean length of admission (days)	3.3	2.2	5.5	0.9	3.4	1.8	2.0	2.8
	Most prevalent ICD-10 chapters in cluster								
	Chapter number: Condition (% of cluster)								
	1^stA^	18: Symptoms/Signs (46)	11: Digestive (46)	5: Mental/Behavioural (100)	15: Pregnancy (100)	18: Symptoms/Signs (93)	19: Injuries/Poisoning (100)	15: Pregnancy (100.0)	18: Symptoms/Signs (84)
	2^nd^	19: Injuries/Poisonings (45)	18: Symptoms/Signs (36)	19: Injury/Poisoning (65)	11: Digestive (19)	19: Injury/Poisoning (89)	13: Musculoskeletal (15)	18: Symptoms/Signs (72)	11: Digestive (79)
	3^rd^	11: Digestive (48)	19: Injury/Poisoning (29)	18: Symptoms/Signs (53)	18: Symptoms/Signs (17)	11: Digestive (88)	18: Symptoms/Signs (13)	14: Genitourinary (62)	13: Musculoskeletal (48)
	4^th^	5: Mental/Behavioural (25)	13: Musculoskeletal (24)	11: Digestive (44)	14: Genitourinary (17)	5: Mental/Behavioural (79)	11: Digestive (11)	11: Digestive (56)	9: Circulatory (48)
	5^th^	13: Musculoskeletal (24)	14: Genitourinary (17)	15: Pregnancy (14)	19: Injury/Poisoning (14)	13: Musculoskeletal (45)	10: Respiratory (7)	19: Injury/Poisoning (52)	19: Injury/Poisoning (46)
Sociodemographic	Sex								
	Female	27,439 (42.3)	9,021 (29.0)	3,452 (33.2)	7,340 (100.0)	2,294 (38.8)	547 (15.2)	3,047 (100.0)	1,399 (44.8)
	Male	37,401 (57.7)	22,111 (71.0)	6,891 (66.8)	0 (0.0)	3,625 (61.2)	3,047 (84.8)	0 (0.0)	1,723 (55.2)
	Age in years (at presentation to D&A services)								
	18-30	4,839 (7.5)	2,306 (7.4)	771 (7.5)	732 (10.0)	175 (3.0)	523 (14.6)	281 (8.3)	51 (1.6)
	31-45	21,856 (33.7)	8,756 (28.1)	3,855 (37.3)	4,125 (56.1)	1,338 (22.6)	1,650 (45.9)	1,751 (51.4)	381 (12.2)
	46-60	29,168 (45.0)	14,945 (48.0)	4,561 (44.2)	2,471 (33.6)	3,073 (51.9)	1,201 (33.4)	1,354 (39.7)	1,563 (50.1)
	60+	8,977 (13.8)	5,125 (16.5)	1,129 (10.9)	22 (0.3)	1,333 (22.5)	220 (6.1)	21 (0.6)	1,127 (36.1)
	Deprivation (IMD) Quintile								
	First (Most deprived)	16,953 (26.7)	7,804 (25.5)	2,968 (29.5)	1,734 (23.9)	1,762 (30.4)	965 (28.1)	911 (27.1)	809 (26.2)
	Second	16,756 (26.4)	7,926 (25.9)	2,709 (26.9)	1,969 (27.1)	1,577 (27.2)	903 (26.3)	922 (27.4)	750 (24.3)
	Third	13,604 (21.4)	6,715 (22.0)	2,049 (20.4)	1,560 (21.5)	1,149 (19.8)	748 (21.8)	675 (20.1)	708 (23.0)
	Fourth	10,901 (17.1)	5,402 (17.7)	1,609 (16.0)	1,324 (18.2)	893 (15.4)	562 (16.3)	561 (16.7)	550 (17.8)
	Fifth (Least deprived)	5,351 (8.4)	2,707 (8.9)	732 (7.3)	672 (9.3)	415 (7.2)	261 (7.6)	296 (8.7)	268 (8.7)
	Residential status								
	Non NFA postcode	63,908 (98.6)	30,700 (98.6)	10,117 (98.0)	7,301 (99.3)	5,843 (98.7)	3,456 (96.2)	3,382 (99.3)	3,109 (99.6)
	NFA postcode	932 (1.4)	432 (1.4)	199 (1.9)	49 (0.7)	76 (1.3)	138 (3.8)	25 (0.7)	13 (0.4)
	Ethnicity^2^								
	White	59,371 (93.8)	28,685 (94.3)	9,201 (91.8)	6,776 (94.3)	5,350 (93.2)	3,298 (94.1)	3,157 (95.0)	2,904 (94.9)
	Non-white	3,900 (6.2)	1,744 (5.7)	825 (8.2)	412 (5.7)	390 (6.8)	207 (5.9)	165 (5.0)	157 (5.1)

Emboldened percentages are statistically significant (p < 0.05) from multivariable, multinomial logistic regression with robust standard errors (Cluster 1: referent) Relative Risk Ratios and 95% confidence intervals can be found in the online supplementary material as table S12; AC1-7 Alcohol Cluster 1-7; D&A Drug and Alcohol; IMD Indices of Multiple Deprivation; NFA No fixed abode; ICD-10 International Classification of Disease-Volume 10; 1 General inpatient admissions are row percentages, all other percentages in table are column percentages 2 Office of Population Censuses and Surveys (OPCS) categories A, B and C collapsed as white, all other OPCS categories (D-S) collapsed as non-white;

**Table 4:** Characteristics of the cohort and diagnostic sub-populations of the *n* = 107,296 individuals who presented to community drug and alcohol treatment services in England in 2018/19 with problems related to opioids.

		All	OC1n (%)	OC2n (%)	OC3n (%)	OC4n (%)	OC5n (%)	OC6n (%)	OC7n (%)
		n (%)	n (%)	n (%)	n (%)	n (%)	n (%)	n (%)	n (%)
All	People n (%)	107,296 (100.0)	50,325 (100.0)	17,910 (100.0)	10,866 (100.0)	8,501 (100.0)	9,442 (100.0)	4,897 (100.0)	5,355 (100.0)
	Mean number of admissions per person (n)	5.2	2.4	6.7	2.6	19.3	1.9	10.0	11.0
	Mean age at first admission (years)	34.0	34.9	32.5	27.9	36.5	30.3	29.1	35.9
	Mean length of admission (days)	4.3	2.4	9.0	1.0	4.1	1.9	2.1	3.6
	Most prevalent ICD-10 chapters in cluster								
	Chapter number: Condition (% of cluster)								
	1^st^	19: Injury/Poisoning (45)	11: Digestive (41)	19: Injury/Poisoning (64)	15: Pregnancy (100)	18: Symptoms/Signs (93)	19: Injury/Poisoning (100)	18: Symptoms/Signs (78)	19: Injury/Poisoning (78)
	2^nd^	18: Symptoms/Signs (40)	18: Symptoms/Signs (30)	5: Mental/Behavioural (54)	11: Digestive (17)	19: Injury/Poisoning (83)	13: Musculoskeletal (14)	15: Pregnancy (66)	12: Skin (77)
	3^rd^	11: Digestive (39)	19: Injury/Poisoning (24)	18: Symptoms/Signs (47)	18: Symptoms/Signs (16)	11: Digestive (79)	11: Digestive (10)	14: Genitourinary (60)	9: Circulatory (71)
	4^th^	13: Musculoskeletal (22)	13: Musculoskeletal (21)	11: Digestive (34)	14: Genitourinary (15)	5: Mental/Behavioural (54)	12: Skin (9)	11: Digestive (57)	13: Musculoskeletal (58)
	5^th^	15: Pregnancy (20)	10: Respiratory (16)	10: Respiratory (18)	19: Injury/Poisoning (10)	10: Respiratory (53)	10: Respiratory (7)	19: Injury/Poisoning (44)	18: Symptoms/Signs (58)
Sociodemographic	Sex								
	Female	31,760 (29.6)	7,883 (15.7)	4,075 (22.7)	10,866 (100.0)	2,695 (31.7)	552 (5.9)	4,645 (94.9)	1,053 (19.7)
	Male	75,536 (70.4)	42,442 (84.3)	13,835 (77.3)	0 (0.0)	5,806 (68.3)	8,890 (94.1)	252 (5.1)	4,302 (80.3)
	Age in years (at presentation to D&A services)								
	18-30	8,036 (7.5)	3,513 (7.0)	1,549 (8.7)	1,095 (10.1)	358 (4.2)	970 (10.3)	431 (8.8)	120 (2.2)
	31-45	58,631 (54.6)	25,061 (49.8)	10,064 (56.2)	7,807 (71.8)	3,612 (42.5)	5,938 (62.9)	3,248 (66.3)	2,901 (54.2)
	46-60	37,110 (34.6)	19,565 (38.9)	5,905 (33.0)	1,951 (18.0)	3,939 (46.3)	2,391 (25.3)	1,171 (23.9)	2,188 (40.9)
	60+	3,519 (3.3)	2,186 (4.3)	329 (2.2)	13 (0.1)	592 (7.0)	143 (1.5)	47 (1.0)	146 (2.7)
	Deprivation (IMD) Quintile								
	First (Most deprived)	38,059 (37.7)	17,152 (36.1)	6,420 (38.7)	3,793 (36.6)	3,280 (40.7)	3,265 (38.0)	1,866 (39.6)	2,283 (44.4)
	Second	28,628 (28.4)	13,620 (28.7)	4,699 (28.3)	2,972 (28.7)	2,281 (28.3)	2,399 (27.9)	1,257 (26.7)	1,400 (27.2)
	Third	18,024 (17.8)	8,700 (18.3)	2,905 (17.5)	1,893 (18.3)	1,374 (17.0)	1,517 (17.7)	857 (18.2)	778 (15.1)
	Fourth	11,533 (11.4)	5,657 (11.9)	1,847 (11.1)	1,190 (11.4)	799 (9.9)	987 (11.4)	540 (11.5)	513 (10.0)
	Fifth (Least deprived)	4,714 (4.7)	2,366 (5.0)	724 (4.4)	508 (5.0)	330 (4.1)	426 (5.0)	193 (4.1)	167 (3.3)
	Residential status								
	Non NFA postcode	100,235 (93.4)	47,141 (93.7)	16,455 (91.9)	10,284 (94.6)	8,041 (94.6)	8,485 (89.9)	4,694 (95.9)	5,135 (95.9)
	NFA postcode	7,061 (6.6)	3,184 (6.3)	1,455 (8.1)	582 (5.4)	460 (5.4)	957 (10.1)	203 (4.1)	220 (4.1)
	Ethnicity^2^								
	White	97,006 (91.8)	45,039 (90.9)	16,049 (91.1)	10,203 (95.3)	7,776 (93.3)	8,358 (90.0)	4,603 (95.5)	4,978 (94.4)
	Non-white	8,615 (8.2)	4,536 (9.1)	1,575 (8.9)	500 (4.7)	562 (6.7)	930 (10.0)	218 (4.5)	294 (5.6)

Emboldened percentages are statistically significant (*p* < 0.05) from multivariable, multinomial logistic regression with robust standard errors (Class 1: referent); Relative Risk Ratios and 95% confidence intervals can be found in the online supplementary material as table S13; D&A Drug and Alcohol; IMD Indices of Multiple Deprivation; NFA No fixed abode; ICD-10 International Classification of Disease-Volume 10; OC1-7 Opioid Cluster 1-7; 1 General inpatient admissions are row percentages, all other percentages in table are column percentages 2 Office of Population Censuses and Surveys (OPCS) categories A, B and C collapsed as white, all other OPCS categories (D-S) collapsed as non-white.

The alcohol and opioid solutions were remarkably similar in composition. Alcohol cluster one (AC1) represented just under half (49.4%) of individuals, comprising predominantly male and older individuals with diseases of the digestive system (46% of cluster) and problems related to injuries or poisoning (29%), but with few individuals admitted due to mental and behavioural problems (3%). Similarly, opioid cluster one (OC1) represented just over two fifths (43.8%) of individuals, comprising predominantly male and older individuals with digestive problems (41%) and problems related to injuries or poisoning (24%), but with few individuals admitted due to mental and behavioural problems (1%).

AC2 represented just over one in ten (13.0%) of individuals, comprising individuals who were from less deprived areas and more likely to be non-white compared with AC1, and had diagnoses predominantly relating to mental and behavioural disorders (100% of cluster) and injuries or poisoning (65%). Similarly, OC2 represented just over one in five (20.8%) of individuals, comprising individuals who were from less deprived areas, and younger compared with OC1, and had diagnoses predominantly relating to injuries or poisoning (64%), and mental and behavioural disorders (54%).

AC3 represented just under one in ten (9.2%) of individuals, comprising exclusively women of childbearing age, who had diagnoses predominantly relating to pregnancy (100% of cluster), and the digestive system (19%). Similarly, OC3 represented just under one in ten (8.2%) of individuals, and were exclusively women of childbearing age, with diagnoses predominantly related to pregnancy (100%) and digestive problems (17%).

AC4 represented just over one in ten (10.3%) of individuals, comprising predominantly older, less affluent men with problems relating to injuries and poisoning (89% of cluster) and digestive problems (88%) but also a high preponderance of admissions due to mental and behavioural disorders (79%). Similarly, OC4 represented just under one in ten (8.3%) of individuals, comprising older more deprived men, who had diagnoses predominantly relating to injuries and poisoning (83%) and digestive problems (79%) but also a high preponderance of admissions due to mental and behavioural disorders (54%).

AC5 represented just over one in twenty (5.6%) of individuals, comprising predominantly younger men with injuries and poisonings (100% of cluster) and musculoskeletal issues (15%). Similarly, OC5 represented just over one in twenty (7%) of individuals, comprising predominantly younger men with injuries and poisonings (100%) and musculoskeletal issues (14%).

AC6 represented just over one in twenty (5.2%) of individuals, comprising exclusively of women of childbearing age who had diagnoses predominantly relating to pregnancy (100% of cluster), and the genitourinary system (62%). Similarly, OC6 represented just over one in twenty (6%) of individuals, comprising mostly of women of childbearing age who had diagnoses predominantly relating to pregnancy (66%), and the genitourinary system (60%).

AC7 represented just over one in twenty (7.0%) of individuals, comprising predominantly older women who had diagnoses predominantly relating to the digestive system (79%) and musculoskeletal problems (48%). OC7 was dissimilar and represented just over one in twenty (5.7%) of individuals, comprising predominantly more deprived men who had diagnoses predominantly relating to injuries and poisoning (78%) and skin problems (77%).

### 3.3 Sociodemographic and clinical associations with rate of inpatient hospital admission, odds of death during treatment and odds of successful treatment completion

Results for the sociodemographic and clinical associations with hospitalisation rate, odds of death during treatment and successful treatment completion can be found in Tables 5 and 6.

**Table 5:** Incidence rate of hospital admissions since 1st April 1997 of the *n* = 64,840 individuals who presented to community drug and alcohol treatment services in England in 2018/19 with problems related to alcohol.

		Peoplen (%)	Admissionsn (%)	Admission rate (Mean number of admissions per person)	AdmissionsaI RR^1^ (95%CI)	p value	Deaths in treatment n (%)	Death in treatment rate (Mean number of deaths per person)	Death in treatmenta OR (95%CI)	p value	People successfully completing treatmentn (%)	Successful treatment rate (Mean number of successful treatments per person)	Successful treatmenta OR (95%CI)	p value
All	All	64,840 (100.0)	374,713 (100.0)	5.8			1,115 (100.0)	0.017			35,581 (100.0)	0.549		
Sociodemographic	Sex													
	Female	27,439 (42.3)	168,490 (45.0)	6.1	Reference		398 (35.7)	0.015	Reference		15,553 (43.7)	0.567	Reference	
	Male	37,401 (57.7)	206,223 (55.0)	5.5	0.87 (0.85-0.90)	<0.001	717 (64.3)	0.019	1.28 (1.11-1.48)	0.001	20,028 (56.3)	0.535	0.89 (0.86-0.93)	<0.001
	Age in years (at presentation to D&A services)													
	18-30	4,839 (7.5)	22,238 (5.9)	4.6	Reference		30 (2.7)	0.001	Reference		2,504 (7.0)	0.517	Reference	
	31-45	21,856 (33.7)	120,866 (32.3)	5.5	1.15 (1.11-1.20)	<0.001	284 (25.5)	0.013	1.99 (1.35-2.92)	<0.001	11,399 (32.0)	0.522	1.03 (0.96-1.10)	0.436
	46-60	29,168 (45.0)	171,898 (45.9)	5.9	1.19 (1.15-1.24)	<0.001	581 (52.1)	0.020	2.78 (1.91-4.06)	<0.001	15,957 (44.9)	0.547	1.13 (1.06-1.21)	<0.001
	60+	8,977 (13.8)	59,711 (15.9)	6.7	1.36 (1.30-1.43)	<0.001	220 (19.7)	0.025	3.25 (2.19-4.82)	<0.001	5,721 (16.1)	0.637	1.70 (1.57-1.84)	<0.001
	Deprivation (IMD) Quintile													
	First (Most deprived)	16,953 (26.7)	111,242 (30.4)	6.6	Reference		301 (27.6)	0.018	Reference		8,824 (25.2)	0.520	Reference	
	Second	16,756 (26.4)	97,204 (26.5)	5.8	0.95 (0.91-0.98)	0.002	279 (25.6)	0.017	0.94 (0.79-1.11)	0.486	9,233 (26.3)	0.551	1.15 (1.09-1.21)	<0.001
	Third	13,604 (21.4)	74,816 (20.4)	5.5	0.94 (0.91-0.98)	0.001	235 (21.5)	0.017	0.97 (0.80-1.16)	0.703	7,575 (21.6)	0.557	1.24 (1.17-1.30)	<0.001
	Fourth	10,901 (17.1)	56,612 (15.4)	5.2	0.91 (0.88-0.95)	<0.001	180 (16.5)	0.017	0.95 (0.78-1.15)	0.588	6,290 (17.9)	0.577	1.33 (1.26-1.41)	<0.001
	Fifth (Least deprived)	5,351 (8.4)	26,665 (7.3)	5.0	0.94 (0.88-0.99)	0.034	94 (8.6)	0.018	1.00 (0.78-1.28)	0.995	3,134 (9.0)	0.586	1.43 (1.33-1.54)	<0.001
	Residential status													
	Non NFA postcode	63,908 (98.6)	368,986 (98.5)	5.8	Reference		1,100 (98.7)	0.017	Reference		35,262 (99.1)	0.594	Reference	
	NFA postcode	932 (1.4)	5,727 (1.5)	6.1	0.82 (0.67-1.02)	0.072	15 (1.3)	0.016	1.11 (0.35-3.50)	0.861	319 (0.9)	0.342	0.49 (0.35-0.68)	<0.001
	Ethnicity^2^													
	White	59,371 (93.8)	342,185 (93.7)	5.8	Reference		1,049 (96.5)	0.018	Reference		32,476 (93.5)	0.547	Reference	
	Non-white	3,900 (6.2)	23,014 (6.3)	5.9	0.94 (0.90-0.98)	0.008	38 (3.5)	0.010	0.56 (0.40-0.77)	0.001	2,245 (6.5)	0.576	1.10 (1.02-1.18)	0.014
Clinical	Diagnostic Cluster													
	AC1	31,132 (48.0)	79,430 (21.2)	2.6	Reference		451 (40.5)	0.014	Reference		17,568 (49.4)	0.564	Reference	
	AC5	3,594 (5.5)	6,269 (1.7)	1.7	0.35 (0.33-0.38)	<0.001	48 (4.3)	0.013	1.01 (0.74-1.39)	0.931	1,893 (5.3)	0.527	0.95 (0.88-1.02)	0.154
	AC3	7,350 (11.3)	19,663 (5.2)	2.7	1.00 (0.96-1.04)	0.931	64 (5.7)	0.009	0.84 (0.63-1.13)	0.257	4,170 (11.7)	0.567	1.01 (0.95-1.08)	0.719
	AC2	10,316 (15.9)	81,559 (21.8)	7.9	2.32 (2.24-2.40)	<0.001	211 (18.9)	0.020	1.56 (1.31-1.85)	<0.001	5,262 (14.8)	0.510	0.81 (0.78-0.86)	<0.001
	AC6	3,407 (5.3)	31,732 (8.5)	9.3	3.01 (2.88-3.15)	<0.001	53 (4.8)	0.016	1.46 (1.06-2.00)	0.019	1,882 (5.3)	0.552	0.97 (0.89-1.05)	0.459
	AC7	3,122 (4.8)	30,534 (8.1)	9.8	4.60 (4.21-5.02)	<0.001	58 (5.2)	0.019	1.21 (0.91-1.60)	0.192	1,873 (5.3)	0.600	1.04 (0.96-1.13)	0.318
	AC4	5,919 (9.1)	125,526 (33.5)	21.2	7.06 (6.72-7.42)	<0.001	230 (20.6)	0.039	2.71 (2.29-3.20)	<0.001	2,933 (8.2)	0.496	0.72 (0.68-0.76)	<0.001

Emboldened percentages are statistically significant (*p* < 0.05) from zero truncated negative binomial regression with robust standard errors; aIRR Adjusted Incidence Rate Ratio; D&A Drug and Alcohol; IMD Indices of Multiple Deprivation; NFA No fixed abode; AC1-7 Alcohol Cluster 1-7; 1 Adjusted for all other covariates listed in table; 2 Office of Population Censuses and Surveys (OPCS) categories A, B and C collapsed as white, all other OPCS categories (D-S) collapsed as non-white.

**Table 6:** Incidence rate of hospital admissions since 1st April 1997 of the *n* = 107,296 individuals who presented to community drug and alcohol treatment services in England in 2018/19 with problems related to opioids.

		Peoplen (%)	Admissionsn (%)	Admission rate (Mean number of admissions per person)	Admissionsa IRR^1^ (95%CI)	p value	Deaths in treatmentn (%)	Death in treatment rate (Mean number of deaths per person)	Death in treatmenta OR (95%CI)	p value	People successfully completing treatmentn (%)	Successful treatment rate (Mean number of successful treatments per person)	Successful treatmenta OR (95%CI)	p value
All	All	107,296 (100)	554,936 (100.0)	5.2			3,301 (100.0)	0.031			12,140 (100.0)	0.113		
Sociodemographic	Sex													
	Female	31,760 (29.6)	205,444 (37.0)	6.5	Reference		874 (26.5)	0.028	Reference		3,836 (31.6)	0.121	Reference	
	Male	75,536 (70.4)	349,492 (63.0)	4.6	0.71 (0.69-0.74)	<0.001	2,427 (73.5)	0.032	1.02 (0.93-1.12)	0.702	8,304 (68.4)	0.110	0.92 (0.87-0.97)	0.002
	Age in years (at presentation to D&A services)													
	18-30	8,036 (7.5)	37,048 (6.7)	4.6	Reference		72 (2.2)	0.009	Reference		1,267 (10.4)	0.158	Reference	
	31-45	58,631 (54.6)	299,491 (54.0)	5.1	1.09 (1.05-1.14)	<0.001	1,230 (37.3)	0.021	2.23 (1.74-2.87)	<0.001	6,322 (52.1)	0.108	0.64 (0.60-0.69)	<0.001
	46-60	37,110 (34.6)	198,835 (35.8)	5.4	1.18 (1.12-1.23)	<0.001	1,692 (51.2)	0.046	4.58 (3.56-5.89)	<0.001	4,063 (33.5)	0.109	0.63 (0.59-0.68)	<0.001
	60+	3,519 (3.3)	19,562 (3.5)	5.6	1.52 (1.42-1.63)	<0.001	307 (9.3)	0.087	8.86 (6.72-11.66)	<0.001	488 (4.0)	0.139	0.79 (0.70-0.88)	<0.001
	Deprivation (IMD) Quintile													
	First (Most deprived)	38,059 (37.7)	210,557 (40.6)	5.5	Reference		1,302 (41.0)	0.034	Reference		3,725 (31.8)	0.098	Reference	
	Second	28,628 (28.4)	146,543 (28.2)	5.1	0.96 (0.94-0.99)	0.008	925 (29.1)	0.032	0.97 (0.89-1.07)	0.565	3,320 (28.3)	0.125	1.13 (1.07-1.19)	<0.001
	Third	18,024 (17.8)	86,184 (16.6)	4.8	0.94 (0.91-0.97)	<0.001	504 (15.9)	0.028	0.84 (0.75-0.94)	0.002	2,308 (19.7)	0.128	1.28 (1.20-1.26)	<0.001
	Fourth	11,533 (11.4)	54,851 (10.6)	4.8	0.95 (0.91-0.99)	0.016	306 (9.6)	0.027	0.78 (0.68-0.90)	<0.001	1,561 (13.3)	0.135	1.34 (1.25-1.44)	<0.001
	Fifth (Least deprived)	4,714 (4.7)	20,984 (4.0)	4.5	0.92 (0.87-0.97)	0.005	140 (4.4)	0.030	0.90 (0.75-1.09)	0.303	799 (6.8)	0.169	1.70 (1.54-1.87)	<0.001
	Residential status													
	Non NFA postcode	100,235 (93.4)	514,512 (92.7)	5.1	Reference		3,178 (96.3)	0.032	Reference		11,697 (96.4)	0.117	Reference	
	NFA postcode	7,061 (6.6)	40,424 (7.3)	5.7	0.94 (0.85-1.03)	0.197	123 (3.7)	0.017	0.76 (0.52-1.11)	0.159	443 (3.6)	0.063	0.47 (0.38-0.58)	<0.001
	Ethnicity^2^													
	White	97,006 (91.8)	506,145 (92.8)	5.2	Reference		3,138 (96.9)	0.032	Reference		10,718 (89.9))	0.110	Reference	
	Non-white	8,615 (8.2)	39,267 (7.2)	4.6	0.98 (0.92-1.04)	0.457	101 (3.1)	0.012	0.36 (0.29-0.44)	<0.001	1,203 (10.1)	0.140	1.32 (1.23-1.42)	<0.001
Clinical	Diagnostic Cluster													
	OC1	50,325 (46.9)	118,299 (21.3)	2.4	Reference		1,467 (44.4)	0.029	Reference		5,785 (47.7)	0.115	Reference	
	OC5	9,442 (8.8)	17,605 (3.2)	1.9	0.42 (0.41-0.44)	<0.001	169 (5.1)	0.018	0.72 (0.61-0.86)	<0.001	972 (8.0)	0.103	0.89 (0.82-0.96)	0.002
	OC3	10,866 (10.1)	28,118 (5.1)	2.6	0.94 (0.90-0.99)	0.01	184 (5.6)	0.017	0.74 (0.62-0.89)	0.001	1,297 (10.7)	0.119	0.97 (0.89-1.05)	0.405
	OC2	17,910 (16.7)	119,200 (21.5)	6.7	2.02 (1.96-2.08)	<0.001	535 (16.2)	0.030	1.14 (1.02-1.26)	0.018	2,049 (16.9)	0.114	1.02 (0.96-1.07)	0.573
	OC6	4,897 (4.6)	48,782 (8.8)	10.0	3.21 (3.05-3.39)	<0.001	110 (3.3)	0.022	0.91 (0.73-1.12)	0.369	642 (5.3)	0.131	1.09 (0.99-1.22)	0.068
	OC7	5,355 (5.0)	58,682 (10.6)	11.0	3.72 (3.59-3.85)	<0.001	292 (8.8)	0.055	1.86 (1.62-2.12)	<0.001	411 (3.4)	0.077	0.69 (0.62-0.77)	<0.001
	OC4	8,501 (7.9)	164,250 (29.6)	19.3	7.50 (7.11-7.91)	<0.001	544 (16.5)	0.064	2.11 (1.90-2.35)	<0.001	984 (8.1)	0.116	1.05 (0.97-1.13)	0.245

Emboldened percentages are statistically significant (*p* < 0.05) from zero truncated negative binomial regression with robust standard errors; aIRR Adjusted Incidence Rate Ratio; D&A Drug and Alcohol; IMD Indices of Multiple Deprivation; NFA No fixed abode; OC1-7 Opioid cluster 1-7; 1 Adjusted for all other covariates listed in table; 2 Office of Population Censuses and Surveys (OPCS) categories A, B and C collapsed as white, all other OPCS categories (D-S) collapsed as non-white.

In the adjusted model, for each substance we found a statistically significantly higher rate of general hospital admissions for women, people aged over 30, people who are from more deprived areas, and people in both alcohol and opioid diagnostic clusters 2, 4, 6, and 7.

In the adjusted model, for each substance we found a statistically significant increase in the odds of death during treatment for older people and people of a white ethnicity and people in both alcohol and opioid diagnostic clusters 2, and 4 and additionally AC6 and OC7.

In the adjusted model, for each substance we found a statistically significantly reduced odds of successful treatment completion for men, people from more deprived areas, people with no fixed residential address and people of a white ethnicity. In addition, there was a reduced odds of successful treatment completion for people in diagnostic clusters AC2, AC4, OC5 and OC7.

No associations changed substantially following inverse probability weighting, suggesting they were not driven by bias from linkage error.

## 4 Discussion

This study represents the first national examination of the hospitalisation of people with problematic alcohol and opioid use presenting to specialist addiction services in England. We identified that 64,840 alcohol patients and 107,296 opioid patients accessing treatment in 2018/19 were responsible for 374,713, and 554,936 general inpatient hospital admissions since 1st April 1997 respectively. Within both these cohorts, women, people over 30, and those residing in more deprived areas have statistically significantly higher admission rates compared to their counterparts. The most common primary admission reason was alcohol withdrawal syndrome (5.3%) in alcohol patients or an unspecified illness (2.1%) in opioid patients. The largest diagnostic clusters of individuals with either substance have a primary admission reason relating to diseases of the digestive system or to injuries and poisonings, but minimal recorded reasons due to mental and behavioural disorders. Following adjustment for sociodemographic characteristics, several diagnostic clusters demonstrated statistically significantly higher hospital admission rates, significantly increased odds of death during treatment and a significantly reduced odds of successful treatment completion for both substances, particularly for those diagnostic clusters in which admission diagnoses predominantly related to mental and behavioural disorders, and injuries or poisonings.

Individuals within these clusters have poor health outcomes and place high cost burdens on healthcare systems through repeated hospital admission. Identification of these individuals, within either addiction or hospital services, may enable more intense personalised treatment, prioritised liaison with specialist mental health services or improved risk management with regards intentional poisoning. If individual services were able to identify the local burden of theses clusters, they may be able to better gauge the level of need within their area, and thus more appropriately plan and budget.

By virtue of these cohorts’ attendance at community addiction services with an alcohol or opioid problem they, by definition, meet ICD-10 diagnostic criteria for at least harmful use, with many likely to meet diagnostic criteria for dependence. As these individuals presented to specialist addiction services in 2018/19 some of their hospital admissions examined herein are likely to pre-date the onset of their problematic substance use, which may lead to conservative estimates of the studied associations, and may partially explain the relative paucity of admissions primarily due to mental and behavioural disorders in each substance's largest diagnostic cluster. However this paucity may also reflect a lack of identification or in-hospital diagnosis that a substance was the likely causative factor in these admissions, which could, in turn, limit the ability of hospital staff to deliver substance-specific interventions to tackle the underlying modifiable risk factor responsible for hospital admission. For example if opioid dependence is never identified as the reason for admission, no consideration would be given to the initiation of opioid substitution treatment (OST), or to the provision of take-home naloxone, and as such the individual may be discharged at a higher risk of death than on admission due to their potentially reduced opioid tolerance.[33]

### 4.1 Strengths and limitations

This study represents the first time that linked data have been interrogated to examine the hospitalisation patterns in a national cohort of individuals presenting to specialist addiction services in England. Inverse probability weighting suggests that the associations reported do not appear to be driven by bias from linkage error. Despite this, there are a number of limitations to consider. Given the observed decline in the numbers of adults entering specialist treatment [10]. and the overall disinvestment and change in the treatment provider landscape since the passage of the Health and Social Care Act in 2012 [34,35], previous work suggests the clinical profile of individuals presenting to services may be shifting from more complex and severe dependence towards lower risk profiles [36]. As such the cohorts described in this study are likely to be only representative of individuals presenting to addiction services in the year studied. There are limitations to using administrative data to examine clinical populations, primarily as coding practices can vary over time and between individual coders. Indeed, over the studied timeframe financial incentives or ‘Payment by Results’ were implemented to incentivise hospitals to diagnose certain conditions [37], including many alcohol attributable conditions, which may have affected the completeness of diagnostic coding. However as the diagnostic clusters are derived from primary admission diagnosis, a field which has been historically well completed, the diagnostic cluster accuracy should be minimally affected by temporal changes in coding practice [38]. The NDTMS categorisation of individuals based on their presenting problematic substance use also means the data used herein are from either patients whom, at presentation in 2018/19, reported that alcohol is the only substance they use problematically or patients whom reported any problematic use of opioids, thus the studied alcohol cohort results cannot be generalised to individuals with any comorbid drug and alcohol problems, and the studied opioid cohort may have individuals who use a variety of other substances in addition to opioids. The nature of this study additionally does not examine the hospitalisation patterns of those individuals with problematic alcohol or opioid use who do not present to publicly funded specialist addiction services. Nevertheless, as both NDTMS and HES APC represent the only national level datasets relating to community addiction treatment and inpatient hospitalisation their linkage, and analysis of the resultant linked data, remains useful to examine national hospitalisation patterns of these individuals. Due to the study's observational nature, there remains the possibility of residual confounding.

## 5 Conclusions

Problematic alcohol and opioid users are highly multimorbid cohorts, with identifiable modifiable risk factors. These individuals place a high cost burden on the healthcare system each year [39], a large proportion of which is due to the use of general inpatient hospital beds. These annual costs look set to increase given national increases in substance-related hospital admissions [6], and significant reductions to real term funding of specialist addiction services [34]. As such, innovative methods need to be found to identify individuals with high hospital usage, in order for clinical teams working in general hospitals and addiction services to develop and target appropriate interventions to both improve their health outcomes and prevent unnecessary readmission. We have aimed to characterise the hospitalisation patterns of this cohort and future work should focus on targeting our identified clusters of individuals with high cost (i.e. high hospital admission rates), high risk of death during treatment, and low odds of successful treatment completion. These are largely characterised by hospital admissions due to mental and behavioural disorders and injuries and poisonings, and previous work has highlighted the lack of integration and barriers between mental health and addiction services in England [36]. As these individuals may be able to be identified using hospital admission patterns prior to engagement with addiction services these results may ultimately help identify individuals in whom problematic alcohol or opioid use was missed, and enable more assertive referral into addiction services [1]. Linkage of further years of NDTMS to HES and analysis of how rates of hospitalisation compare over time would be useful next steps to identify potential mechanisms to reduce unnecessary readmission, and additionally prioritisation of further work with cohorts of people not accessing services could improve the lives of people suffering from substance use disorders.

## Contributors

All authors meet the ICMJE criteria for authorship:

Dr Roberts formulated the research question, designed and carried out the study, analysed the data and drafted the article

Professor Hotopf contributed to the formulation of the research question, study design, data analysis and writing the article

Professor Marsden contributed to the data interpretation and writing the article

Professor Strang contributed to the data interpretation and writing the article

Mr White contributed to the study design, data analysis and writing the article

Dr Eastwood contributed to the formulation of the research question, study design, data analysis and writing the article

Professor Drummond contributed to the formulation of the research question, study design, data analysis and writing the article

Dr Roberts has nothing to disclose

Professor Hotopf reports grants from Innovative Medicines Initiative, outside the submitted work.

Professor Strang reports other from Molteni Farma, grants from Mundipharma, grants from Camurus, other from Accord Pharma, outside the submitted work.

Professor Marsden reports grants from National Institute for Health Research (NIHR), grants from NIHR Biomedical Research Centre for Mental Health at South London and Maudsley NHS Mental Health Foundation Trust (SLaM), grants from Indivior, outside the submitted work; and I have part-time employment as Senior Academic Advisor for the Alcohol, Drugs, Tobacco and Justice Division, Health Improvement, Public Health England and I am a clinical academic consultant for the US National Institute on Drug Abuse, Centre for Clinical Trials Network. I have received honoraria and travel support from PCM Scientific and Martindale for the Improving Outcomes in Treatment of Opioid Dependence conference. I hold no stocks in any company.

Mr White has nothing to disclose

Dr Eastwood has nothing to disclose

Professor Drummond has nothing to disclose

## Data sharing statement

Whilst access to the linked dataset is only available within Public Health England, subject to approval, extracts of NDTMS are available to researchers through the Office of Data Release (ODR) at PHE, and extracts of HES APC are available through the Data Access Request Service (DARS) at NHS Digital. In addition, the code for the linkage algorithm will also be made available upon request to PHE, subject to approval.

## Role of the funding source

This paper represents independent research funded by the Medical Research Council (MRC), as part of the corresponding author's MRC Addiction Research Clinical (MARC) Fellowship. The research was part funded by the NIHR Biomedical Research Centre at South London and Maudsley NHS Foundation Trust and King's College London, and by the NIHR Collaboration for Leadership in Applied Health Research and Care South London (NIHR CLAHRC South London) now recommissioned as NIHR Applied Research Collaboration South London, and both CD, MH and JS receive funding from an NIHR Senior Investigator award. The funders had no contribution to the study design; in the collection, analysis, and interpretation of data; in the writing of the report; and in the decision to submit the article for publication. All authors were independent from funders had full access to all of the data (including statistical reports and tables) in the study and take responsibility for the integrity of the data and the accuracy of the data analysis. The views expressed are those of the authors and not necessarily those of the MRC, the National Health Service (NHS), the NIHR, Public Health England (PHE) or the Department of Health and Social Care (DHSC).

## Declaration of Competing Interest

All Individual authors have completed the ICJME Conflict of Interest form:

## Appendix Supplementary materials

Image, application 1
