# Supplementary material for: The hospital admission profile of people presenting to specialist addiction services with problematic use of alcohol or opioids: A national retrospective cohort study in England
Source: Lancet Reg Health Eur. 2021 Jan 17;3:100036. doi: 10.1016/j.lanepe.2021.100036 (PMC8080891; doi:10.1016/j.lanepe.2021.100036)
Supplement: Supplementary file 1 [file mmc1.docx]

**Online Supplementary Material**

Contents

Tables

S1: ICD-10 Chapters

S2: Top ten primary reasons for general inpatient admissions restricted to three-digit ICD-10 code level since 1^st^ April 1997 of the individuals who presented to substance misuse treatment services in England in 2018/19

S3: Goodness of fit of latent class models for the n=64,840 people with problematic use of alcohol

S4: Goodness of fit of latent class models for the n=107,296 people with problematic use of opioids

S5: The proportion of individuals in each cluster within the whole cohort, and the proportion of people whom had ever had an admission from each ICD-10 chapter per cluster for the n=64,840 people with problematic use of alcohol

S6: The proportion of individuals in each cluster within the whole cohort, and the proportion of people whom had ever had an admission from each ICD-10 chapter per cluster for the n=107,296 people with problematic use of opioids

S7: Top ten primary reasons for general inpatient admissions per cluster since 1^st^ April 1997 of the individuals who presented to substance misuse treatment services in England in 2018/19 for the n=64,840 people with problematic use of alcohol

S8: Top ten primary reasons for general inpatient admissions per cluster since 1^st^ April 1997 of the individuals who presented to substance misuse treatment services in England in 2018/19 for the n=107,296 people with problematic use of opioids

S9: STROBE checklist for cohort studies

S10: Variables used in multivariable analysis

S11: STROBE checklist for conference abstracts (2)

S12: Multinomial logistic regression with robust standard errors (Cluster AC1: referent) of the n=64,840 individuals who presented to community drug and alcohol treatment services in England in 2018/19 with problems related to alcohol

S13: Multinomial logistic regression with robust standard errors (Cluster OC1: referent) of the n=107,296 individuals who presented to community drug and alcohol treatment services in England in 2018/19 with problems related to opioids

Figures

S1: Participant flow diagram

Comments

S1: Latent Class Analysis (LCA) model specifications

S2: Statistical and clinical assessments of Latent Class Analysis (LCA) clusters

References

**Tables**

Table S1: ICD-10 Chapters (1)

| **Chapter** | **Disease Classification** | **Examples** |
| --- | --- | --- |
| 1 | Certain infectious and parasitic diseases | e.g., Bacterial, viral, protozoal diseases |
| 2 | Neoplasms | e.g., Malignant and benign cancerous diseases |
| 3 | Diseases of the blood and blood-forming organs and certain disorders involving the immune mechanism | e.g., Anaemias and coagulation disorders |
| 4 | Endocrine, nutritional and metabolic diseases | e.g., Diabetes mellitus, thyroid and metabolic diseases |
| 5 | Mental and behavioural disorders | e.g., Mood, anxiety, psychotic and substance use disorders |
| 6 | Diseases of the nervous system | e.g., Central and peripheral nervous system disorders |
| 7 | Diseases of the eye and adnexa | e.g., Visual disturbances and glaucoma |
| 8 | Diseases of the ear and mastoid process | e.g., Disorders or the external, inner and middle ear |
| 9 | Diseases of the circulatory system | e.g., Ischaemic and hypertensive heart disease |
| 10 | Diseases of the respiratory system | e.g., Respiratory infection, diseases of the pleura |
| 11 | Diseases of the digestive system | e.g., Liver, biliary, stomach and intestinal disease |
| 12 | Diseases of the skin and subcutaneous tissue | e.g., Dermatitis, skin and soft tissue infections |
| 13 | Diseases of the musculoskeletal system and connective tissue | e.g., Arthritis and systemic connective tissue disorders |
| 14 | Diseases of the genitourinary system | e.g., Renal and bladder disorders |
| 15 | Pregnancy, childbirth and the puerperium | e.g., Pregnancy and labour related disorders |
| 16 | Certain conditions originating in the perinatal period | e.g., Birth trauma and disorders of the newborn |
| 17 | Congenital malformations, deformations and chromosomal abnormalities | e.g., Congenital malformations including cleft lip and palate |
| 18 | Symptoms, signs and abnormal clinical and laboratory findings, not elsewhere classified | e.g., Abnormal findings on examination or investigation |
| 19 | Injury, poisoning and certain other consequences of external causes | e.g., Injuries, burns, poisonings and frostbite |
| 20* | External causes of morbidity and mortality | e.g., Accidents and exposure related problems |
| 21* | Factors influencing health status and contact with health services | e.g., Genetic carriers, socioeconomic circumstances |
| 22* | Codes for special purposes | e.g., Diseases of uncertain aetiology or emergency use |

^* Not included in the latent class models^

Table S2: Top ten primary reasons for general inpatient admissions restricted to three-digit ICD-10 code level since 1^st^ April 1997 of the individuals who presented to substance misuse treatment services in England in 2018/19

| Rank of Admission | **Primary reason for admission**  **(three-digit ICD-10 code level)** | **ICD-10 chapter** | **ICD-10**  **code** | **Admissions in substance cohort**  n (%) | **Admissions in all HES**  n (%) |
| --- | --- | --- | --- | --- | --- |
| Alcohol |  |  |  |  |  |
| All | All | All | All | 374,713 (100.0) | 157,885,932 (100.0) |
| 1 (Most common) | Mental and behavioural disorders due to alcohol | 5 | F10 | 43,047 (11.5) | 880,794 (0.6) |
| 2 | Abdominal and pelvic pain | 18 | R10 | 13,633 (3.6) | 4,693,035 (3.0) |
| 3 | Poisoning by, adverse effect of and underdosing of nonopioid analgesics, antipyretics and antirheumatics | 19 | T39 | 12,802 (3.4) | 955,971 (0.6) |
| 4 | Pain in throat and chest | 18 | R07 | 11,986 (3.2) | 4,923,926 (3.1) |
| 5 | Poisoning by, adverse effect of and underdosing of psychotropic drugs, not elsewhere classified | 19 | T43 | 7,557 (2.0) | 437,391 (0.3) |
| 6 | Illness, unspecified | 18 | R69 | 6,558 (1.8) | 2,662,891 (1.7) |
| 7 | Gastrointestinal hemorrhage, unspecified | 11 | K92 | 6,142 (1.6) | 996,540 (0.6) |
| 8 | Alcoholic liver disease | 11 | K70 | 6,124 (1.6) | 286,091 (0.2) |
| 9 | Maternal care for other conditions predominantly related to pregnancy | 15 | O26 | 5,535 (1.5) | 2,314,624 (1.5) |
| 10 (Least common) | Poisoning by, adverse effect of and underdosing of antiepileptic, sedative- hypnotic and antiparkinsonism drugs | 19 | T42 | 4,990 (1.3) | 321,694 (0.2) |
| Opioid |  |  |  |  |  |
| All | All | All | All | 554,936 (100.0) | 157,885,932 (100.0) |
| 1 (Most common) | Abdominal and pelvic pain | 18 | R10 | 21,491 (3.9) | 4,693,035 (3.0) |
| 2 | Mental and behavioural disorders due to alcohol | 5 | F10 | 17,537 (3.2) | 880,794 (0.6) |
| 3 | Pain in throat and chest | 18 | R07 | 15,478 (2.8) | 4,923,926 (3.1) |
| 4 | Cutaneous abscess, furuncle and carbuncle | 12 | L02 | 13,756 (2.5) | 610,457 (0.4) |
| 5 | Poisoning by, adverse effect of and underdosing of narcotics and psychodysleptics [hallucinogens] | 19 | T40 | 13,008 (2.3) | 243,958 (0.2) |
| 6 | Poisoning by, adverse effect of and underdosing of nonopioid analgesics, antipyretics and antirheumatics | 19 | T39 | 12,187 (2.2) | 955,971 (0.6) |
| 7 | Illness, unspecified | 18 | R69 | 11,387 (2.1) | 2,662,891 (1.7) |
| 8 | Mental and behavioural disorders due to opioids | 5 | F11 | 11,011 (1.98) | 60,025 (0.04) |
| 9 | Cellulitis and acute lymphangitis | 12 | L03 | 10,527 (1.9) | 1,308,574 (0.8) |
| 10 (Least common) | Phlebitis and thrombophlebitis of other and unspecified deep vessels of lower extremities | 9 | I80 | 10,520 (1.9) | 568,763 (0.4) |

^ICD-10 international Classification of Diseases Volume 10^

Table S3: Goodness of fit of latent class models for the n=64,840 people with problematic use of alcohol

|  | One cluster | Two clusters | Three clusters | Four clusters | Five clusters | Six clusters | Seven clusters* | Eight clusters |
| --- | --- | --- | --- | --- | --- | --- | --- | --- |
| Expected percentage of individuals per cluster (%) | C1: 100.0 | C1: 75.5 | C1: 47.3 | C1: 11.1 | C1: 9.0 | C1: 9.3 | C1: 49.5 | C1: 11.9 |
|  |  | C2: 24.5 | C2: 30.2 | C2: 37.6 | C2: 37.5 | C2: 45.2 | C2: 13.0 | C2: 6.2 |
|  |  |  | C3: 22.5 | C3: 30.0 | C3: 29.9 | C3: 16.5 | C3: 9.2 | C3: 39.8 |
|  |  |  |  | C4: 21.3 | C4: 6.2 | C4: 12.2 | C4: 10.3 | C4: 9.4 |
|  |  |  |  |  | C5: 17.4 | C5: 5.7 | C5: 5.6 | C5: 10.3 |
|  |  |  |  |  |  | C6: 11.1 | C6: 5.2 | C6: 12.0 |
|  |  |  |  |  |  |  | C7: 7.1 | C7: 4.4 |
|  |  |  |  |  |  |  |  | C8: 6.0 |
| Likelihood Ratio (LR) test vs saturated model (p) | 1.00 | 1.00 | 1.00 | 1.00 | 1.00 | 1.00 | 1.00 | 1.00 |
| Entropy | - | 0.689 | 0.408 | 0.642 | 0.653 | 0.661 | 0.875 | 0.897 |
| Akaike's Information Criterion (AIC) | 949988 | 921935 | 918058 | 915026 | 913447 | 911933 | 911155 | 910703 |
| Bayesian Information Criteria (BIC) | 950161 | 922289 | 918593 | 915725 | 914328 | 912995 | 912317 | 912010 |

* Chosen as the LCA model

Table S4: Goodness of fit of latent class models for the n=107,296 people with problematic use of opioids

|  | One cluster | Two clusters | Three clusters | Four clusters | Five clusters | Six clusters | Seven clusters* | Eight clusters |
| --- | --- | --- | --- | --- | --- | --- | --- | --- |
| Expected percentage of individuals per cluster (%) | C1: 100.0 | C1: 79.2 | C1: 40.7 | C1: 6.3 | C1: 7.3 | C1: 7.4 | C1: 43.8 | C1: 8.7 |
|  |  | C2: 20.8 | C2: 39.8 | C2: 40.3 | C2: 32.6 | C2: 34.8 | C2: 20.8 | C2: 9.1 |
|  |  |  | C3: 19.5 | C3: 34.5 | C3: 39.8 | C3: 35.4 | C3: 8.3 | C3: 6.7 |
|  |  |  |  | C4: 18.9 | C4: 13.8 | C4: 6.0 | C4: 8.2 | C4: 47.9 |
|  |  |  |  |  | C5: 6.5 | C5: 11.3 | C5: 6.8 | C5: 6.3 |
|  |  |  |  |  |  | C6: 5.1 | C6: 6.3 | C6: 6.0 |
|  |  |  |  |  |  |  | C7: 5.7 | C7: 10.9 |
|  |  |  |  |  |  |  |  | C8: 4.3 |
| Likelihood Ratio (LR) test vs saturated model (p) | 1.0 | 1.0 | 1.0 | 1.0 | 1.0 | 1.0 | 1.0 | 1.0 |
| Entropy | - | 0.704 | 0.364 | 0.621 | 0.635 | 0.631 | 0.880 | 0.863 |
| Akaike's Information Criterion (AIC) | 1471929 | 1433436 | 1426255 | 1422885 | 1419344 | 1417652 | 1415133 | 1413882 |
| Bayesian Information Criteria (BIC) | 1472111 | 1433810 | 1426820 | 1423623 | 1420273 | 1418764 | 1416388 | 1415358 |

* Chosen as the LCA model

Table S5: The proportion of individuals in each cluster within the whole cohort, and the proportion of people whom had ever had an admission from each ICD-10 chapter per cluster for the n=64,840 people with problematic use of alcohol

|  |  | Cluster one (AC1) | | Cluster two (AC2) | | Cluster three (AC3) | | Cluster four (AC4) | | Cluster five (AC5) | | Cluster six (AC6) | | Cluster seven (AC7) | |
| --- | --- | --- | --- | --- | --- | --- | --- | --- | --- | --- | --- | --- | --- | --- | --- |
|  |  | Expected percentage of people per cluster (95% CI) | 49.5  (48.2-50.8) | Expected percentage of people per cluster  (95% CI) | 13.0  (12.2-13.9) | Expected percentage of people per cluster  (95% CI) | 9.2  (8.5-9.9) | Expected percentage of people per cluster  (95% CI) | 10.3  (9.1-11.7) | Expected percentage of people per cluster  (95% CI) | 5.6  (5.0-6.3) | Expected percentage of people per cluster  (95% CI) | 5.2  (4.7-5.9) | Expected percentage of people per cluster (95% CI) | 7.1  (5.9-8.5) |
| Chapter Name | Chapter Number | Marginal probability of having admission within this chapter | Chapter Rank | Marginal probability of having admission within this chapter | Chapter Rank | Marginal probability of having admission within this chapter | Chapter Rank | Marginal probability of having admission within this chapter | Chapter Rank | Marginal probability of having admission within this chapter | Chapter Rank | Marginal probability of having admission within this chapter | Chapter Rank | Marginal probability of having admission within this chapter | Chapter Rank |
| Certain infectious and parasitic diseases | 1 | 0.049 | 13 | 0.045 | 14 | 0.013 | 13 | 0.300 | 9 | 0.008 | 13 | 0.123 | 10 | 0.207 | 11 |
| Neoplasms | 2 | 0.136 | 8 | 0.065 | 12 | 0.073 | 6 | 0.197 | 13 | 0.000 | 18 | 0.166 | 9 | 0.402 | 7 |
| Diseases of the blood and blood-forming organs and disorders involving the immune mechanism | 3 | 0.018 | 17 | 0.018 | 16 | 0.012 | 14 | 0.125 | 15 | 0.000 | 19 | 0.033 | 15 | 0.161 | 13 |
| Endocrine, nutritional and metabolic diseases | 4 | 0.030 | 15 | 0.046 | 13 | 0.013 | 12 | 0.218 | 12 | 0.004 | 15 | 0.068 | 13 | 0.164 | 12 |
| Mental and behavioural disorders | 5 | 0.033 | 14 | 1.000 | 1 | 0.000 | 19 | 0.785 | 4 | 0.053 | 8 | 0.239 | 6 | 0.150 | 14 |
| Diseases of the nervous system | 6 | 0.061 | 11 | 0.067 | 11 | 0.023 | 11 | 0.248 | 10 | 0.019 | 10 | 0.100 | 12 | 0.226 | 10 |
| Diseases of the eye and adnexa | 7 | 0.059 | 12 | 0.037 | 15 | 0.012 | 15 | 0.106 | 16 | 0.015 | 11 | 0.027 | 16 | 0.146 | 15 |
| Diseases of the ear and mastoid process | 8 | 0.023 | 16 | 0.015 | 17 | 0.010 | 16 | 0.031 | 17 | 0.013 | 12 | 0.025 | 17 | 0.043 | 17 |
| Diseases of the circulatory system | 9 | 0.151 | 6 | 0.105 | 8 | 0.024 | 10 | 0.405 | 7 | 0.000 | 17 | 0.064 | 14 | 0.475 | 4 |
| Diseases of the respiratory system | 10 | 0.138 | 7 | 0.123 | 7 | 0.065 | 7 | 0.427 | 6 | 0.074 | 5 | 0.211 | 8 | 0.343 | 8 |
| Diseases of the digestive system | 11 | 0.458 | 1 | 0.441 | 4 | 0.193 | 2 | 0.877 | 3 | 0.110 | 4 | 0.564 | 4 | 0.787 | 2 |
| Diseases of the skin and subcutaneous tissue | 12 | 0.108 | 9 | 0.086 | 10 | 0.033 | 9 | 0.234 | 11 | 0.064 | 7 | 0.102 | 11 | 0.241 | 9 |
| Diseases of the musculoskeletal system and connective tissue | 13 | 0.235 | 4 | 0.139 | 6 | 0.053 | 8 | 0.446 | 5 | 0.151 | 2 | 0.235 | 7 | 0.483 | 3 |
| Diseases of the genitourinary system | 14 | 0.173 | 5 | 0.103 | 9 | 0.168 | 4 | 0.363 | 8 | 0.025 | 9 | 0.624 | 3 | 0.445 | 6 |
| Pregnancy, childbirth and the puerperium | 15 | 0.078 | 10 | 0.144 | 5 | 1.000 | 1 | 0.133 | 14 | 0.068 | 6 | 1.000 | 1 | 0.111 | 16 |
| Certain conditions originating in the perinatal period | 16 | 0.002 | 19 | 0.000 | 19 | 0.000 | 18 | 0.002 | 19 | 0.001 | 16 | 0.002 | 19 | 0.000 | 19 |
| Congenital malformations, deformations and chromosomal abnormalities | 17 | 0.015 | 18 | 0.007 | 18 | 0.007 | 17 | 0.019 | 18 | 0.005 | 14 | 0.015 | 18 | 0.035 | 18 |
| Symptoms, signs and abnormal clinical and laboratory findings, not elsewhere classified | 18 | 0.356 | 2 | 0.533 | 3 | 0.172 | 3 | 0.934 | 1 | 0.128 | 3 | 0.720 | 2 | 0.835 | 1 |
| Injury, poisoning and certain other consequences of external causes | 19 | 0.288 | 3 | 0.645 | 2 | 0.144 | 5 | 0.892 | 2 | 1.000 | 1 | 0.521 | 5 | 0.456 | 5 |
|  | Average | 0.127 | - | 0.190 | - | 0.106 | - | 0.355 | - | 0.091 | - | 0.255 | - | 0.301 | - |
|  | Cluster Name^1^ | 11,18,19,13,14 | | 5,19,18,11,15 | | 15,11,18,14,19 | | 18,19,11,5,13 | | 19,13,18,11,10 | | 15,18,14,11,19 | | 18,11,13,9,19 | |
|  | Cluster Description | Largely digestive and injury/poisoning diagnoses with few admissions due to mental and behavioural disorders due to alcohol and predominantly male, predominantly older | | Largely mental and behavioural disorders due to alcohol and injuries/poisoning. More affluent, and more non-white individuals compared to cluster one | | Exclusively women of childbearing age, largely diagnoses relating to pregnancy and the digestive system. More affluent compared to cluster one | | Largely digestive problems and problems relating to injuries and poisoning but also a high preponderance of issues due to mental and behavioural disorders due to alcohol. Older, male and less affluent compared to cluster one | | Largely injuries, poisonings and musculoskeletal issues, younger and more male compared to cluster one | | Exclusively women of childbearing age who had diagnoses predominantly relating to pregnancy, and the genitourinary system. More affluent comparted to cluster one | | Largely musculoskeletal and digestive problems. More women and older people compared to cluster one | |

^1 Based on the top five most prevalent clusters^

Table S6: The proportion of individuals in each cluster within the whole cohort, and the proportion of people whom had ever had an admission from each ICD-10 chapter per cluster for the n=107,296 people with problematic use of opioids

|  |  | Cluster one (OC1) | | Cluster two (OC2) | | Cluster three (OC3) | | Cluster four (OC4) | | Cluster five (OC5) | | Cluster six (OC6) | | Cluster seven (OC7) | |
| --- | --- | --- | --- | --- | --- | --- | --- | --- | --- | --- | --- | --- | --- | --- | --- |
|  |  | Expected percentage of people per cluster (95% CI) | 43.8  (42.1-45.6) | Expected percentage of people per cluster  (95% CI) | 20.8  (19.1-22.7) | Expected percentage of people per cluster  (95% CI) | 8.2  (7.7-8.7) | Expected percentage of people per cluster  (95% CI) | 8.3  (7.8-8.9) | Expected percentage of people per cluster  (95% CI) | 6.8  (6.3-7.3) | Expected percentage of people per cluster  (95% CI) | 6.3  (5.6-7.0) | Expected percentage of people per cluster (95% CI) | 5.7  (5.3-6.2) |
| Chapter Name | Chapter Number | Marginal probability of having admission within this chapter | Chapter Rank | Marginal probability of having admission within this chapter | Chapter Rank | Marginal probability of having admission within this chapter | Chapter Rank | Marginal probability of having admission within this chapter | Chapter Rank | Marginal probability of having admission within this chapter | Chapter Rank | Marginal probability of having admission within this chapter | Chapter Rank | Marginal probability of having admission within this chapter | Chapter Rank |
| Certain infectious and parasitic diseases | 1 | 0.06 | 11 | 0.06 | 11 | 0.01 | 13 | 0.34 | 10 | 0.01 | 8 | 0.14 | 11 | 0.25 | 9 |
| Neoplasms | 2 | 0.10 | 9 | 0.03 | 13 | 0.07 | 7 | 0.14 | 14 | 0.01 | 12 | 0.21 | 8 | 0.04 | 14 |
| Diseases of the blood and blood-forming organs and disorders involving the immune mechanism | 3 | 0.02 | 16 | 0.01 | 18 | 0.01 | 18 | 0.13 | 15 | 0.00 | 16 | 0.05 | 15 | 0.05 | 13 |
| Endocrine, nutritional and metabolic diseases | 4 | 0.03 | 14 | 0.01 | 15 | 0.01 | 15 | 0.16 | 13 | 0.00 | 16 | 0.05 | 14 | 0.01 | 16 |
| Mental and behavioural disorders | 5 | 0.01 | 18 | 0.54 | 2 | 0.01 | 12 | 0.54 | 4 | 0.00 | 16 | 0.16 | 9 | 0.39 | 6 |
| Diseases of the nervous system | 6 | 0.05 | 12 | 0.05 | 12 | 0.01 | 11 | 0.23 | 11 | 0.00 | 14 | 0.12 | 13 | 0.06 | 12 |
| Diseases of the eye and adnexa | 7 | 0.04 | 13 | 0.02 | 14 | 0.01 | 14 | 0.08 | 16 | 0.01 | 9 | 0.03 | 17 | 0.02 | 15 |
| Diseases of the ear and mastoid process | 8 | 0.02 | 15 | 0.01 | 16 | 0.01 | 16 | 0.03 | 17 | 0.01 | 10 | 0.03 | 16 | 0.01 | 17 |
| Diseases of the circulatory system | 9 | 0.13 | 8 | 0.08 | 9 | 0.02 | 10 | 0.44 | 7 | 0.02 | 7 | 0.13 | 12 | 0.71 | 3 |
| Diseases of the respiratory system | 10 | 0.16 | 5 | 0.18 | 5 | 0.08 | 6 | 0.53 | 5 | 0.07 | 5 | 0.27 | 6 | 0.38 | 7 |
| Diseases of the digestive system | 11 | 0.41 | 1 | 0.34 | 4 | 0.17 | 2 | 0.79 | 3 | 0.10 | 3 | 0.57 | 4 | 0.32 | 8 |
| Diseases of the skin and subcutaneous tissue | 12 | 0.14 | 7 | 0.14 | 6 | 0.04 | 8 | 0.42 | 8 | 0.09 | 4 | 0.14 | 10 | 0.77 | 2 |
| Diseases of the musculoskeletal system and connective tissue | 13 | 0.21 | 4 | 0.13 | 7 | 0.04 | 9 | 0.48 | 6 | 0.14 | 2 | 0.27 | 7 | 0.58 | 4 |
| Diseases of the genitourinary system | 14 | 0.14 | 6 | 0.07 | 10 | 0.15 | 4 | 0.36 | 9 | 0.04 | 6 | 0.60 | 3 | 0.10 | 11 |
| Pregnancy, childbirth and the puerperium | 15 | 0.08 | 10 | 0.12 | 8 | 1.00 | 1 | 0.18 | 12 | 0.00 | 16 | 0.66 | 2 | 0.12 | 10 |
| Certain conditions originating in the perinatal period | 16 | 0.00 | 19 | 0.00 | 19 | 0.00 | 19 | 0.00 | 19 | 0.00 | 15 | 0.00 | 19 | 0.00 | 19 |
| Congenital malformations, deformations and chromosomal abnormalities | 17 | 0.02 | 17 | 0.01 | 17 | 0.01 | 17 | 0.02 | 18 | 0.01 | 11 | 0.03 | 18 | 0.00 | 18 |
| Symptoms, signs and abnormal clinical and laboratory findings, not elsewhere classified | 18 | 0.30 | 2 | 0.47 | 3 | 0.16 | 3 | 0.93 | 1 | 0.01 | 13 | 0.78 | 1 | 0.58 | 5 |
| Injury, poisoning and certain other consequences of external causes | 19 | 0.24 | 3 | 0.64 | 1 | 0.10 | 5 | 0.83 | 2 | 1.00 | 1 | 0.44 | 5 | 0.78 | 1 |
|  | Average | 0.11 | - | 0.15 | - | 0.10 | - | 0.35 | - | 0.08 | - | 0.25 | - | 0.27 | - |
|  | Cluster Name^1^ | 11,18,19,13,10 | | 19,5,18,11,10 | | 15,11,18,14,19 | | 18,19,11,5,10 | | 19,13,11,12,10 | | 18,15,14,11,19 | | 19,12,9,13,18 | |
|  | Cluster Description | Predominantly digestive problems and injuries and poisonings but almost no mental and behavioural disorders | | Predominantly injuries and poisonings and mental and behavioural disorders. Compared to cluster one younger, with a long length of stay and more deprived | | Exclusively women of childbearing age with pregnancy related disorders and digestive problems | | Predominantly injuries and poisonings and digestive problems with a high predominance of mental and behavioural disorders. Compared to cluster one very high admission rate, older and more deprived | | Predominantly injuries and poisonings, musculoskeletal disorders and digestive problems. Compared to cluster one few and short admissions | | Predominantly middle-aged women with disorders relating to pregnancy and genitourinary problems | | People with skin and circulatory conditions Compared to cluster one more deprived middle aged predominantly male | |

^1 Based on the top five most prevalent clusters^

S7: Top ten primary reasons for general inpatient admissions per cluster since 1^st^ April 1997 of the individuals who presented to substance misuse treatment services in England in 2018/19 for the n=64,840 people with problematic use of alcohol

|  | **All alcohol cohort** | | | **AC1** | | | **AC2** | | | **AC3** | | |
| --- | --- | --- | --- | --- | --- | --- | --- | --- | --- | --- | --- | --- |
| Rank of Admission | **Primary reason for admission** | **ICD-10**  **code** | **Admissions in substance cohort**  n (%) | **Primary reason for admission** | **ICD-10**  **code** | **Admissions in substance cohort**  n (%) | **Primary reason for admission** | **ICD-10**  **code** | **Admissions in substance cohort**  n (%) | **Primary reason for admission** | **ICD-10**  **code** | **Admissions in substance cohort**  n (%) |
|  |  | All | 374,713 (100.0) |  | All | 79430 (100) |  | All | 81559 (100) |  | All | 19663 (100) |
| 1 (Most common) | Alcohol withdrawal state | F10.3 | 20,024 (5.3) | Chest pain, unspecified | R074 | 2111 (2.66) | Alcohol withdrawal state | F103 | 9472 (11.61) | Other specified pregnancy related conditions | O268 | 1588 (8.08) |
| 2 | Acute alcohol intoxication | F10.0 | 11,206 (3.0) | Paracetamol poisoning | T391 | 1469 (1.85) | Acute alcohol intoxication | F100 | 5524 (6.77) | Maternal care for other specified fetal problems | O368 | 1209 (6.15) |
| 3 | Paracetamol poisoning | T39.1 | 10,731 (2.9) | Abdominal pain, unspecified | R104 | 1380 (1.74) | Alcohol dependence | F102 | 3933 (4.82) | Encounter for supervision of other than normal pregnancy | Z348 | 708 (3.6) |
| 4 | Alcohol dependence | F10.2 | 8,056 (2.2) | Illness, unspecified | R69 | 1121 (1.41) | Paracetamol poisoning | T391 | 3551 (4.35) | Supervision of normal pregnancy, unspecified | Z349 | 630 (3.2) |
| 5 | Chest pain, unspecified | R07.4 | 6,846 (1.8) | Chest pain, other | R073 | 1026 (1.29) | Illness, unspecified | R69 | 2188 (2.68) | Hemorrhage in early pregnancy, unspecified | O209 | 536 (2.73) |
| 6 | Illness, unspecified | R69 | 6,558 (1.8) | Pain localized to other parts of lower abdomen | R103 | 949 (1.19) | Antidepressant poisoning | T432 | 1651 (2.02) | Antepartum hemorrhage, unspecified | O469 | 501 (2.55) |
| 7 | Abdominal pain, unspecified | R10.4 | 6,240 (1.7) | Headache | R51 | 839 (1.06) | Convulsions, unspecified | R568 | 1259 (1.54) | False labor at or after 37 weeks of gestation | O471 | 377 (1.92) |
| 8 | Other specified pregnancy related conditions | O26.8 | 4,565 (1.2) | Pain localized to upper abdomen | R101 | 750 (0.94) | Other symptoms and signs involving emotional state | R458 | 1186 (1.45) | Paracetamol poisoning | T391 | 365 (1.86) |
| 9 | Antidepressant poisoning | T43.2 | 4,346 (1.2) | Syncope and collapse | R55 | 709 (0.89) | Harmful use of alcohol | F101 | 1080 (1.32) | Other specified diseases complicating pregnancy, childbirth or the puerperium | O998 | 363 (1.85) |
| 10 (Least common) | Convulsions, unspecified | R56.8 | 4,120 (1.1) | Other and unspecified asthma | J459 | 660 (0.83) | Unspecified urinary incontinence | F329 | 1080 (1.32) | Missed abortion | O021 | 345 (1.75) |
|  | **AC4** | | | **AC5** | | | **AC6** | | | **AC7** | | |
| Rank of Admission | **Primary reason for admission** | **ICD-10**  **code** | **Admissions in substance cohort**  n (%) | **Primary reason for admission** | **ICD-10**  **code** | **Admissions in substance cohort**  n (%) | **Primary reason for admission** | **ICD-10**  **code** | **Admissions in substance cohort**  n (%) | **Primary reason for admission** | **ICD-10**  **code** | **Admissions in substance cohort**  n (%) |
|  |  | All | 125526 (100) |  | All | 6269 (100) |  | All | 31732 (100) |  | All | 30534 (100) |
| 1 (Most common) | Alcohol withdrawal state | F103 | 9646 (7.68) | Paracetamol poisoning | T391 | 396 (6.32) | Other specified pregnancy related conditions | O268 | 1666 (5.25) | Chest pain, unspecified | R074 | 978 (3.2) |
| 2 | Acute alcohol intoxication | F100 | 5232 (4.17) | Unspecified injury of the head | S099 | 190 (3.03) | Abdominal pain, unspecified | R104 | 981 (3.09) | Abdominal pain, unspecified | R104 | 522 (1.71) |
| 3 | Paracetamol poisoning | T391 | 3933 (3.13) | Fracture of the mandible | S026 | 153 (2.44) | Pain localized to other parts of lower abdomen | R103 | 864 (2.72) | Chest pain, other | R073 | 431 (1.41) |
| 4 | Alcohol dependence | F102 | 3741 (2.98) | Fracture of the lower end of radius | S525 | 149 (2.38) | Maternal care for other specified fetal problems | O368 | 853 (2.69) | Illness, unspecified | R69 | 394 (1.29) |
| 5 | Chest pain, unspecified | R074 | 2515 (2) | Antidepressant poisoning | T432 | 145 (2.31) | Paracetamol poisoning | T391 | 824 (2.6) | Pain localized to other parts of lower abdomen | R103 | 391 (1.28) |
| 6 | Abdominal pain, unspecified | R104 | 2255 (1.8) | Fracture of other parts of lower leg | S828 | 122 (1.95) | Alcohol withdrawal state | F103 | 670 (2.11) | Pain localized to upper abdomen | R101 | 334 (1.09) |
| 7 | Haematemesis | K920 | 2124 (1.69) | Open wound of thumb without damage to nail | S610 | 107 (1.71) | Illness, unspecified | R69 | 545 (1.72) | Urinary tract infection, site not specified | N390 | 325 (1.06) |
| 8 | Illness, unspecified | R69 | 2098 (1.67) | Open wound of other parts of hand | S018 | 94 (1.5) | Supervision of normal pregnancy, unspecified | Z349 | 529 (1.67) | Headache | R51 | 293 (0.96) |
| 9 | Convulsions, unspecified | R568 | 2014 (1.6) | Fracture of other and unspecified finger(s) | S626 | 75 (1.2) | Hemorrhage in early pregnancy, unspecified | O209 | 493 (1.55) | Other and unspecified asthma | J459 | 289 (0.95) |
| 10 (Least common) | Syncope and collapse | R55 | 1704 (1.36) | Fracture of malar, maxillary and zygoma bones | S024 | 70 (1.12) | Encounter for supervision of other than normal pregnancy | Z348 | 449 (1.41) | Unspecified acute lower respiratory infection | J22 | 270 (0.88) |

S8: Top ten primary reasons for general inpatient admissions per cluster since 1^st^ April 1997 of the individuals who presented to substance misuse treatment services in England in 2018/19 for the n=107,296 people with problematic use of opioids

|  | **All opioid cohort** | | | **OC1** | | | **OC2** | | | **OC3** | | |
| --- | --- | --- | --- | --- | --- | --- | --- | --- | --- | --- | --- | --- |
| Rank of Admission | **Primary reason for admission** | **ICD-10**  **code** | **Admissions in substance cohort**  n (%) | **Primary reason for admission** | **ICD-10**  **code** | **Admissions in substance cohort**  n (%) | **Primary reason for admission** | **ICD-10**  **code** | **Admissions in substance cohort**  n (%) | **Primary reason for admission** | **ICD-10**  **code** | **Admissions in substance cohort**  n (%) |
|  | All | All | 554,936 (100.0) | All | All | 118299 (100) | All | All | 119200 (100) | All | All | 28118 (100) |
| 1 (Most common) | Illness, unspecified | R69 | 11,387 (2.1) | Chest pain, unspecified | R074 | 2488 (2.1) | Opioid dependence | F112 | 4858 (4.08) | Other specified pregnancy related conditions | O268 | 2350 (8.36) |
| 2 | Abdominal pain, unspecified | R10.4 | 10,589 (1.9) | Abdominal pain, unspecified | R104 | 2161 (1.83) | Illness, unspecified | R69 | 4474 (3.75) | Maternal care for other specified fetal problems | O368 | 1821 (6.48) |
| 3 | Paracetamol poisoning | T39.1 | 9,744 (1.8) | Other and unspecified asthma | J459 | 1792 (1.51) | Paracetamol poisoning | T391 | 3620 (3.04) | Encounter for supervision of other than normal pregnancy | Z348 | 1018 (3.62) |
| 4 | Cellulitis and acute lymphangitis of other parts of limb | L03.1 | 9,078 (1.6) | Cellulitis and acute lymphangitis of other parts of limb | L031 | 1787 (1.51) | Paranoid schizophrenia | F200 | 3052 (2.56) | Supervision of normal pregnancy, unspecified | Z349 | 884 (3.14) |
| 5 | Chest pain, unspecified | R07.4 | 8,719 (1.6) | Illness, unspecified | R69 | 1526 (1.29) | Acute alcohol intoxication | F100 | 2701 (2.27) | Hemorrhage in early pregnancy, unspecified | O209 | 779 (2.77) |
| 6 | Opioid dependence | F11.2 | 8,625 (1.6) | Phlebitis and thrombophlebitis of other and unspecified deep vessels of lower extremities | I802 | 1508 (1.27) | Alcohol dependence | F102 | 2647 (2.22) | Antepartum hemorrhage, unspecified | O469 | 760 (2.7) |
| 7 | Other specified pregnancy related conditions | O26.8 | 7,890 (1.4) | Lobar pneumonia, unspecified organism | J181 | 1505 (1.27) | Benzodiazepine poisoning | T424 | 2468 (2.07) | Other specified diseases complicating pregnancy, childbirth or the puerperium | O998 | 574 (2.04) |
| 8 | Phlebitis and thrombophlebitis of other and unspecified deep vessels of lower extremities | I80.2 | 7,498 (1.4) | Cutaneous abscess, furuncle and carbuncle of limb | L024 | 1410 (1.19) | Harmful use of alcohol | F103 | 2398 (2.01) | Encounter for supervision of normal first pregnancy | Z340 | 517 (1.84) |
| 9 | Cutaneous abscess, furuncle and carbuncle of limb | L02.4 | 6,616 (1.2) | Chest pain, other | R073 | 1386 (1.17) | Heroin poisoning | T401 | 1933 (1.62) | False labor at or after 37 weeks of gestation | O471 | 468 (1.66) |
| 10 (Least common) | Benzodiazepine poisoning | T42.4 | 6,473 (1.2) | Pain localized to other parts of abdomen | R103 | 1291 (1.09) | Abdominal pain, unspecified | R104 | 1751 (1.47) | Missed abortion | O021 | 465 (1.65) |
|  | **OC5** | | | **OC6** | | | **OC7** | | | **OC8** | | |
| Rank of Admission | **Primary reason for admission** | **ICD-10**  **code** | **Admissions in substance cohort**  n (%) | **Primary reason for admission** | **ICD-10**  **code** | **Admissions in substance cohort**  n (%) | **Primary reason for admission** | **ICD-10**  **code** | **Admissions in substance cohort**  n (%) | **Primary reason for admission** | **ICD-10**  **code** | **Admissions in substance cohort**  n (%) |
|  |  | All | 164250 (100) |  | All | 17605 (100) |  | All | 48782 (100) |  | All | 58682 (100) |
| 1 (Most common) | Abdominal pain, unspecified | R104 | 3978 (2.42) | Unspecified injury of the head | S099 | 543 (3.08) | Other specified pregnancy related conditions | O268 | 2815 (5.77) | Phlebitis and thrombophlebitis of other and unspecified deep vessels of lower extremities | I802 | 3715 (6.33) |
| 2 | Chest pain, unspecified | R074 | 3436 (2.09) | Paracetamol poisoning | T391 | 513 (2.91) | Abdominal pain, unspecified | R104 | 1883 (3.86) | Cellulitis and acute lymphangitis of other parts of limb | L031 | 3262 (5.56) |
| 3 | Illness, unspecified | R69 | 3392 (2.07) | Heroin poisoning | T401 | 479 (2.72) | Maternal care for other specified fetal problems | O368 | 1411 (2.89) | Cutaneous abscess, furuncle and carbuncle of trunk | L022 | 2440 (4.16) |
| 4 | Paracetamol poisoning | T391 | 3081 (1.88) | Fracture of mandible | S026 | 474 (2.69) | Pain localized to other parts of lower abdomen | R103 | 1378 (2.82) | Cutaneous abscess, furuncle and carbuncle of limb | L024 | 2082 (3.55) |
| 5 | Cellulitis and acute lymphangitis of other parts of limb | L031 | 2752 (1.68) | Fracture of the lower end of radius | S525 | 322 (1.83) | Illness, unspecified | R69 | 832 (1.71) | Other specified soft tissue disorders | M798 | 1627 (2.77) |
| 6 | Alcohol withdrawal state | F103 | 2464 (1.5) | Benzodiazepine poisoning | T424 | 319 (1.81) | Paracetamol poisoning | T391 | 774 (1.59) | Opioid dependence | F112 | 1367 (2.33) |
| 7 | Opioid dependence | F112 | 2133 (1.3) | Open wound of thumb without damage to nail | S610 | 308 (1.75) | Encounter for supervision of other than normal pregnancy | Z348 | 738 (1.51) | Non pressure chronic ulcer of lower limb | L97 | 1338 (2.28) |
| 8 | Alcohol dependence syndrome | F102 | 2112 (1.29) | Cutaneous abscess, furuncle and carbuncle of limb | L024 | 283 (1.61) | Supervision of normal pregnancy, unspecified | Z349 | 681 (1.4) | Phlebitis and thrombophlebitis of femoral vein | I801 | 1191 (2.03) |
| 9 | Acute alcohol intoxication | F100 | 2073 (1.26) | Cellulitis and acute lymphangitis of other parts of limb | L031 | 270 (1.53) | Hemorrhage in early pregnancy, unspecified | O209 | 676 (1.39) | Heroin poisoning | T401 | 1183 (2.02) |
| 10 (Least common) | Benzodiazepine poisoning | T424 | 2024 (1.23) | Fractures of other parts of lower leg | S828 | 254 (1.44) | Pain localized to upper abdomen | R101 | 650 (1.33) | Illness, unspecified | R69 | 932 (1.59) |

S9: STROBE checklist for cohort studies (2)

|  | Item No | Recommendation |
| --- | --- | --- |
| **Title and abstract** | 1 | (*a*) Indicate the study’s design with a commonly used term in the title or the abstract; Title: Page 1 |
|  |  | (*b*) Provide in the abstract an informative and balanced summary of what was done and what was found; Abstract: Page 3 |
| Introduction | | |
| Background/rationale | 2 | Explain the scientific background and rationale for the investigation being reported; Introduction: Page 6 |
| Objectives | 3 | State specific objectives, including any prespecified hypotheses; Introduction: Page 6 |
| Methods | | |
| Study design | 4 | Present key elements of study design early in the paper; Methods: Pages 8-10 |
| Setting | 5 | Describe the setting, locations, and relevant dates, including periods of recruitment, exposure, follow-up, and data collection; Methods: Pages 8 and 9 |
| Participants | 6 | (*a*) Give the eligibility criteria, and the sources and methods of selection of participants. Describe methods of follow-up; Methods: Pages 8 and 9 |
|  |  | (*b*) For matched studies, give matching criteria and number of exposed and unexposed N/A |
| Variables | 7 | Clearly define all outcomes, exposures, predictors, potential confounders, and effect modifiers. Give diagnostic criteria, if applicable Methods: Pages 9-10 |
| Data sources/ measurement | 8* | For each variable of interest, give sources of data and details of methods of assessment (measurement). Describe comparability of assessment methods if there is more than one group Methods: Pages 9-10 |
| Bias | 9 | Describe any efforts to address potential sources of bias Methods: Pages 9-10 and role of the funding source |
| Study size | 10 | Explain how the study size was arrived at Methods Page 8; Results Page 12 |
| Quantitative variables | 11 | Explain how quantitative variables were handled in the analyses. If applicable, describe which groupings were chosen and why Methods Pages 9 and 10 |
| Statistical methods | 12 | (*a*) Describe all statistical methods, including those used to control for confounding Methods Page 10 |
|  |  | (*b*) Describe any methods used to examine subgroups and interactions Methods Page 10 |
|  |  | (*c*) Explain how missing data were addressed Methods Page 10 |
|  |  | (*d*) If applicable, explain how loss to follow-up was addressed N/A but ‘non-response’ bias methods page 10 and11 |
|  |  | (*e*) Describe any sensitivity analyses methods page 10 |
| Results | | |
| Participants | 13* | (a) Report numbers of individuals at each stage of study—eg numbers potentially eligible, examined for eligibility, confirmed eligible, included in the study, completing follow-up, and analysed Results Page 12 |
|  |  | (b) Give reasons for non-participation at each stage Results page 12 |
|  |  | (c) Consider use of a flow diagram N/A |
| Descriptive data | 14* | (a) Give characteristics of study participants (eg demographic, clinical, social) and information on exposures and potential confounders Results pages 12-13 and Tables 1 |
|  |  | (b) Indicate number of participants with missing data for each variable of interest Table 1 |
|  |  | (c) Summarise follow-up time (eg, average and total amount) Methods page 8 Results page 10 |
| Outcome data | 15* | Report numbers of outcome events or summary measures over time Table 5 and 6 |
| Main results | 16 | (*a*) Give unadjusted estimates and, if applicable, confounder-adjusted estimates and their precision (eg, 95% confidence interval). Make clear which confounders were adjusted for and why they were included Table 5 and 6 |
|  |  | (*b*) Report category boundaries when continuous variables were categorized Table 1 and 5 and 6 |
|  |  | (*c*) If relevant, consider translating estimates of relative risk into absolute risk for a meaningful time period N/A |
| Other analyses | 17 | Report other analyses done—eg analyses of subgroups and interactions, and sensitivity analyses Results Page 15 |
| Discussion | | |
| Key results | 18 | Summarise key results with reference to study objectives Discussion Page 16 |
| Limitations | 19 | Discuss limitations of the study, taking into account sources of potential bias or imprecision. Discuss both direction and magnitude of any potential bias Discussion pages 17 and 18 |
| Interpretation | 20 | Give a cautious overall interpretation of results considering objectives, limitations, multiplicity of analyses, results from similar studies, and other relevant evidence Conclusions page 18 |
| Generalisability | 21 | Discuss the generalisability (external validity) of the study results Discussion Page 17 |
| Other information | | |
| Funding | 22 | Give the source of funding and the role of the funders for the present study and, if applicable, for the original study on which the present article is based Methods Page 11 |

*Give information separately for exposed and unexposed groups

S10: Variables used in multivariate analysis

| **Variable** | **Variable Type** | **Notes** |
| --- | --- | --- |
| Sex | Binary;  1= Female  2= Male | Reference: Female |
| Age | Ordinal; 4 levels  1= 18-30  2= 31-45  3= 46-60  4= 60+ | Reference: 18-30  In years (at presentation to D&A services) |
| Deprivation quintile (using Indices of Multiple Deprivation (IMD)) | Ordinal; 5 levels  1= First (Most deprived)  2= Second  3= Third  4= Fourth  5= Fifth (Least deprived) | Reference: First (Most deprived) |
| Residential Status | Binary;  1= Housed (Non-NFA postcode)  2= Not Housed (NFA-postcode) | Reference: Housed  Record contains either a postcode designated as No Fixed Abode (NFA) or contains any other postcode |
| Ethnicity | Categorical; 16 Categories  1=White British  2=White Irish  3=Any other White background  4=White and Black Caribbean (Mixed)  5=White and Black African (Mixed)  6=White and Asian (Mixed)  7=Any other mixed background  8=Indian (Asian or Asian British)  9=Pakistani (Asian or Asian British)  10=Bangladeshi (Asian or Asian British)  11=Any other Asian background  12=Caribbean (Black or Black British)  13=African (Black or Black British)  14=Any other Black background  Chinese  15=Any other ethnic group  16=Not stated/Not known | Office of Population Censuses and Surveys (OPCS) definitions (3, 4)  Collapsed into binary:  1=White (1-3)  2= Non-white (4-15)  Reference: White |
| Diagnostic cluster | Categorical; 7 categories  1=AC1/OC1  2=AC2/OC2  3=AC3/OC3  4=AC4/OC4  5=AC5/OC5  6=AC6/OC6  7=AC7/OC7 | Reference: AC1/OC1  AC1= Alcohol Cluster One  OC1=Opioid Cluster One etc. |

S11: STROBE checklist for conference abstracts (2)

| **Item** | **Recommendation** |
| --- | --- |
| Title | Indicate the study’s design with a commonly used term in the title (e.g cohort, case-control, cross sectional) Title Page 1 |
| Authors | Contact details for the corresponding author Page 1 |
| Study design | Description of the study design (e.g cohort, case-control, cross sectional) Title Page 1 |
| Objective | Specific objectives or hypothesis Introduction Page 3 |
| Methods | |
| Setting | Description of setting, follow-up dates or dates at which the outcome events occurred or at which the outcomes were present, as well as any points or ranges on other time scales for the outcomes (e.g., prevalence at age 18, 1998-2007). Methods Page 3 |
| Participants | *Cohort study*—Give the most important eligibility criteria, and the most important sources and methods of selection of participants. Describe briefly the methods of follow-up Methods Page 3  *Case-control study*—Give the major eligibility criteria, and the major sources and methods of case ascertainment and control selection  *Cross-sectional study*—Give the eligibility criteria, and the major sources and methods of selection of participants |
| *Cohort study*—For matched studies, give matching and number of exposed and unexposed  *Case-control study*—For matched studies, give matching criteria and the number of controls per case N/A | |
| Variables | Clearly define primary outcome for this report. Methods Page 3 |
| Statistical methods | Describe statistical methods, including those used to control for confounding Methods Page 3 |
| Results | |
| Participants | Report Number of participants at the beginning and end of the study Results Page 3 |
| Main results | Report estimates of associations. If relevant, consider translating estimates of relative risk into absolute risk for a meaningful time period  Report appropriate measures of variability and uncertainty (e.g., odds ratios with confidence intervals Results Page 3 |
| Conclusions | General interpretation of study results Interpretation Page 3 |

S12: Multinomial logistic regression with robust standard errors (Cluster AC1: referent) of the n=64,840 individuals who presented to community drug and alcohol treatment services in England in 2018/19 with problems related to alcohol

|  | AC2  RRR (95%CI) | AC3  RRR (95%CI) | AC4  RRR (95%CI) | AC5  RRR (95%CI) | AC6  RRR (95%CI) | AC7  RRR (95%CI) |
| --- | --- | --- | --- | --- | --- | --- |
| Sex |  |  |  |  |  |  |
| Female | Reference | Reference | Reference | Reference | Reference | Reference |
| Male | **0.76 (0.73-0.81)** | **0.00 (0.00-0.00)** | **0.64 (0.60-0.68)** | **2.08 (1.87-2.30)** | **0.00 (0.00-0.00)** | **0.55 (0.51-0.60)** |
| Age in years (at presentation to D&A services) |  |  |  |  |  |  |
| 18-30 | Reference | Reference | Reference | Reference | Reference | Reference |
| 31-45 | **1.38 (1.25-1.52)** | **2.20 (1.96-2.46)** | **2.08 (1.75-2.49)** | **0.78 (0.70-0.86)** | **2.44 (2.11-2.82)** | **2.21 (1.63-3.00)** |
| 46-60 | 0.93 (0.84-1.03) | **0.56 (0.49-0.64)** | **2.75 (2.33-3.26)** | **0.35 (0.31-0.39)** | **0.82 (0.71-0.95)** | **5.03 (3.73-6.77)** |
| 60+ | **0.68 (0.61-0.77)** | **0.01 (0.01-0.02)** | **3.52 (2.93-4.23)** | **0.19 (0.16-0.23)** | **0.03 (0.02-0.05)** | **10.4 (7.63-14.06)** |
| Deprivation (IMD) Quintile |  |  |  |  |  |  |
| First (Most deprived) | Reference | Reference | Reference | Reference | Reference | Reference |
| Second | **0.90 (0.81-0.99)** | 1.08 (0.98-1.18) | **0.85 (0.77-0.94)** | 0.94 (0.84-1.06) | 0.95 (0.84-1.08) | 0.88 (0.79-0.97) |
| Third | **0.82 (0.74-0.91)** | 1.01 (0.93-1.10) | **0.72 (0.65-0.81)** | 0.96 (0.86-1.07) | **0.82 (0.72-0.93)** | 0.92 (0.82-1.02) |
| Fourth | **0.82 (0.73-0.92)** | 1.05 (0.97-1.14) | **0.68 (0.59-0.78)** | 0.95 (0.83-1.08) | **0.82 (0.72-0.92)** | **0.83 (0.73-0.93)** |
| Fifth (Least deprived) | **0.75 (0.65-0.87)** | 1.06 (0.94-1.20) | **0.62 (0.54-0.72)** | 0.87 (0.75-1.02) | **0.87 (0.75-1.01)** | **0.79 (0.68-0.92)** |
| Residential status |  |  |  |  |  |  |
| Non NFA postcode | Reference | Reference | Reference | Reference | Reference | Reference |
| NFA postcode | 1.04 (0.66-1.63) | 0.55 (0.25-1.24) | 0.89 (0.48-1.67) | 0.96 (0.55-1.66) | 0.82 (0.38-1.74) | 0.16 (0.02-1.04) |
| Ethnicity^1^ |  |  |  |  |  |  |
| White | Reference | Reference | Reference | Reference | Reference | Reference |
| Non-white | **1.42 (1.27-1.58)** | 1.03 (0.89-1.20) | **1.31 (1.15-1.49)** | 0.87 (0.77-1.00) | 0.93 (0.78-1.10) | 1.11 (0.94-1.31) |

Emboldened percentages are statistically significant (p < 0.05 AC2-7 Alcohol Cluster 2-7; D&A Drug and Alcohol; IMD Indices of Multiple Deprivation; NFA No fixed abode; RRR Relative Risk Ratio; CI Confidence Interval; 1 Office of Population Censuses and Surveys (OPCS) categories A, B and C collapsed as white, all other OPCS categories (D-S) collapsed as non-white;

S13: Multinomial logistic regression with robust standard errors (Cluster OC1: referent) of the n=107,296 individuals who presented to community drug and alcohol treatment services in England in 2018/19 with problems related to opioids

|  | OC2  RRR (95%CI) | OC3  RRR (95%CI) | OC4  RRR (95%CI) | OC5  RRR (95%CI) | OC6  RRR (95%CI) | OC7  RRR (95%CI) |
| --- | --- | --- | --- | --- | --- | --- |
| Sex |  |  |  |  |  |  |
| Female | Reference | Reference | Reference | Reference | Reference | Reference |
| Male | **0.60 (0.57-0.63)** | **0.00 (0.00-0.00)** | **0.39 (0.37-0.41)** | **3.20 (2.91-3.51)** | **0.01 (0.01-0.01)** | **0.72 (0.67-0.78)** |
| Age in years (at presentation to D&A services) |  |  |  |  |  |  |
| 18-30 | Reference | Reference | Reference | Reference | Reference | Reference |
| 31-45 | 0.94 (0.87-1.01) | **1.62 (1.47-1.80)** | **1.57 (1.39-1.77)** | **0.77 (0.70-0.84)** | **1.72 (1.52-1.94)** | **3.49 (2.85-4.29)** |
| 46-60 | **0.71 (0.64-0.78)** | **0.53 (0.47-0.60)** | **2.18 (1.90-2.49)** | **0.39 (0.36-0.44)** | **0.81 (0.70-0.93)** | **3.31 (2.65-4.14)** |
| 60+ | **0.42 (0.37-0.48)** | **0.02 (0.01-0.04)** | **2.77 (2.38-3.23)** | **0.22 (0.18-0.27)** | **0.20 (0.15-0.27)** | **2.03 (1.54-2.68)** |
| Deprivation (IMD) Quintile |  |  |  |  |  |  |
| First (Most deprived) | Reference | Reference | Reference | Reference | Reference | Reference |
| Second | 0.93 (0.84-1.03) | 1.03 (0.96-1.11) | **0.88 (0.79-0.98)** | 0.93 (0.87-1.00) | **0.88 (0.80-0.97)** | **0.79 (0.69-0.92)** |
| Third | **0.89 (0.80-0.99)** | 1.00 (0.93-1.07) | **0.82 (0.72-0.92)** | **0.91 (0.84-0.98)** | 0.92 (0.83-1.01) | **0.67 (0.58-0.78)** |
| Fourth | **0.88 (0.77-0.99)** | 0.98 (0.89-1.09) | **0.73 (0.64-0.84)** | 0.91 (0.82-1.01) | 0.90 (0.81-1.01) | **0.68 (0.57-0.81)** |
| Fifth (Least deprived) | **0.82 (0.71-0.95)** | 1.07 (0.90-1.26) | **0.72 (0.61-0.86)** | 0.93 (0.82-1.05) | **0.81 (0.68-0.96)** | **0.53 (0.41-0.69)** |
| Residential status |  |  |  |  |  |  |
| Non NFA postcode | Reference | Reference | Reference | Reference | Reference | Reference |
| NFA postcode | 1.05 (0.92-1.20) | **0.63 (0.52-0.75)** | **0.75 (0.61-0.93)** | **1.30 (1.11-1.53)** | **0.55 (0.41-0.75)** | **0.61 (0.46-0.80)** |
| Ethnicity^1^ |  |  |  |  |  |  |
| White | Reference | Reference | Reference | Reference | Reference | Reference |
| Non-white | 1.04 (0.96-1.14) | 0.90 (0.79-1.03) | **0.78 (0.70-0.87)** | 1.04 (0.96-1.13) | **0.85 (0.73-0.99)** | **0.60 (0.51-0.70)** |

Emboldened percentages are statistically significant (p < 0.05 OC2-7 Opioid Cluster 2-7; D&A Drug and Alcohol; IMD Indices of Multiple Deprivation; NFA No fixed abode; RRR Relative Risk Ratio; CI Confidence Interval; 1 Office of Population Censuses and Surveys (OPCS) categories A, B and C collapsed as white, all other OPCS categories (D-S) collapsed as non-white;

Figures

S1: Participant Flow Diagram

The number of unique people presenting to all publicly funded addiction services, with problematic use of alcohol or opioids, in England in 2018/19:

Unique people with problematic use of alcohol: n=75,555

Unique people with problematic use of opioids: n=139,845

The number of unique people presenting to all publicly funded addiction services, with problematic use of alcohol or opioids, in England in 2018/19 who linked to HES APC hospitalisation records available since 1^st^ April 1997:

Unique people with problematic use of alcohol: n=64,840 (85.8% of the total number of people with problematic use of alcohol presenting to addiction services in England in 2018/19)

Unique people with problematic use of opioids: n=107,296 (76.7% of the total number of people with problematic use of opioids presenting to addiction services in England in 2018/19)

The number of unique hospital admissions, since HES database inception on 1^st^ April 1997, people with problematic use of alcohol or opioids who presented to addiction services in England in 2018/19, and linked to HES APC, were responsible for:

Unique hospital admissions by unique people with problematic use of alcohol: n=374,713

Unique hospital admissions by unique people with problematic use of opioids: n=554,936

Comments

S1: Latent Class Analysis (LCA) model specifications

In order to guard against convergence on local rather than global maxima, we planned to use an iterative maximum likelihood estimate initially with at least 5000 random sets of starting values combined with an inspection of the corresponding log likelihood values. If necessary, we would have increased the number of random sets until the log likelihood had been replicated a minimum of five times, however this was never required as stability was demonstrated on replication with the use of 5000 random sets. The software’s default burn in period was used (2,500 iterations).

S2: Statistical and clinical assessments of Latent Class Analysis (LCA) clusters

The initial statistical and clinical assessments were made by Dr Emmert Roberts (lead author, a medically qualified addiction psychiatrist and epidemiologist and Dr Brian Eastwood (Statistician at Public Health England). Subsequently authors Professors Colin Drummond and Matthew Hotopf (both also medically qualified psychiatrists with epidemiological training) were additionally consulted.

**References**

1. WHO. The ICD-10 classification of mental and behavioural disorders : diagnostic criteria for research. Geneva : World Health Organization. Geneva : World Health Organization. 1993;World Health Organization.

2. Von Elm E, Altman DG, Egger M, Pocock SJ, Gøtzsche PC, Vandenbroucke JP. The Strengthening the Reporting of Observational Studies in Epidemiology (STROBE) statement: guidelines for reporting observational studies. Annals of internal medicine. 2007;147(8):573-7.

3. Harmonised country specific ethnic group question(s) and dissemination of output(s) for use in social surveys and administrative data in England, Northern Ireland, Scotland and Wales <https://gss.civilservice.gov.uk/policy-store/ethnicity/#great-britain>.

4. Hospital Episode Statistics Data Dictionary <https://digital.nhs.uk/data-and-information/data-tools-and-services/data-services/hospital-episode-statistics/hospital-episode-statistics-data-dictionary>.
